# Volatile emission and biosynthesis in endophytic fungi colonizing black poplar leaves

**DOI:** 10.3762/bjoc.17.118

**Published:** 2021-07-22

**Authors:** Christin Walther, Pamela Baumann, Katrin Luck, Beate Rothe, Peter H W Biedermann, Jonathan Gershenzon, Tobias G Köllner, Sybille B Unsicker

**Affiliations:** 1 Department of Biochemistry, Max Planck Institute for Chemical Ecology, Hans-Knöll Str. 8, 07745 Jena, Germany; 2 Chair of Forest Entomology and Protection, Institute of Forest Sciences, University of Freiburg, Fohrenbühl 27, 79252 Stegen-Wittental, Germany

**Keywords:** Ascomycota, *Cladosporium*, Salicaceae, terpene synthases, volatile organic compound (VOC)

## Abstract

Plant volatiles play a major role in plant–insect interactions as defense compounds or attractants for insect herbivores. Recent studies have shown that endophytic fungi are also able to produce volatiles and this raises the question of whether these fungal volatiles influence plant–insect interactions. Here, we qualitatively investigated the volatiles released from 13 endophytic fungal species isolated from leaves of mature black poplar (*Populus nigra*) trees. The volatile blends of these endophytes grown on agar medium consist of typical fungal compounds, including aliphatic alcohols, ketones and esters, the aromatic alcohol 2-phenylethanol and various sesquiterpenes. Some of the compounds were previously reported as constituents of the poplar volatile blend*.* For one endophyte, a species of *Cladosporium*, we isolated and characterized two sesquiterpene synthases that can produce a number of mono- and sesquiterpenes like (*E*)-β-ocimene and (*E*)-β-caryophyllene, compounds that are dominant components of the herbivore-induced volatile bouquet of black poplar trees. As several of the fungus-derived volatiles like 2-phenylethanol, 3-methyl-1-butanol and the sesquiterpene (*E*)-β-caryophyllene, are known to play a role in direct and indirect plant defense, the emission of volatiles from endophytic microbial species should be considered in future studies investigating tree-insect interactions.

## Introduction

Plant volatile organic compounds (VOCs) can mediate plant–insect, plant–microbe, and plant–plant interactions [1–4]. The constitutive and herbivore-induced volatile blends of plants consist of different compound classes, including green leaf volatiles, benzenoids, terpenoids, and nitrogen-containing compounds [5–7]. Among these, terpenoids represent the largest and most diverse group of compounds. In poplar trees, large amounts of terpenoids can be emitted constitutively [8–9] and facilitate protection against thermal and oxidative stresses [10]. In addition, terpenoids are also produced in response to biological stresses such as herbivory [9,11] and can fulfill different functions in plant–insect interactions. For instance, together with other volatiles, some terpenoids are known to attract natural enemies of insect herbivores [2,12–13] or attract insects as shown for the sesquiterpene (*E*)-β-caryophyllene (**1**) [14–15]. Another sesquiterpene, (*E*)-β-farnesene (**2**), an aphid alarm pheromone, is also produced by plant species like *Arabidopsis thaliana* [16]. Besides terpenoids, other plant VOCs are also known to mediate plant–insect interactions. For instance, 2-phenylethanol (**3**) is a typical attractant for pollinators, but is also involved in direct and indirect plant defense [17–19].

Endophytic microorganisms are fungi or bacteria that live asymptomatically within healthy plant tissue (e.g., leaves, flowers and roots) for at least a part of their life cycle [20]. Endophyte colonization is widespread in the plant kingdom, but their role in plant–insect interactions is under debate [21]. Currently, most of our knowledge on the role of endophytes in plant defense responses comes from studies with fungal grass endophytes (clavicipitaceous endophytes) that are often mutualistic for the plant. The ecological significance of nonclavicipitaceous endophytes, which occur also in trees, is more ambiguous and only poorly understood [22–24].

Endophytic fungi themselves can produce VOCs. Currently, around 300 fungal VOCs have been characterized, including aliphatic alcohols, ketones, aldehydes, acids and esters, terpenoids, benzenoids, naphthalene derivatives, and cycloalkanes [25–27]. Endophytic fungal VOCs are frequently described to exhibit antimicrobial activity; however, they are also known to induce the growth and vigor of the host plant and to shape plant community structure [27–31]. Furthermore, volatiles released from endophytic fungi can also affect insect behavior. Daisy et al. isolated the endophytic fungus *Muscodor vitigenus* and characterized the volatile blend in culture [32]. Naphthalene, an insect deterrent that is used, e.g., in mothballs [33], was the most dominant compound in the fungal volatile blend and showed a repellent effect on the wheat stem sawfly *Cephus cinctus* in a Y-tube olfactometer experiment. However, the literature on endophytic volatiles and how they influence insect behavior is scarce, especially for the endophytes of trees despite the omnipresence of fungal endophytes in forest ecosystems [34] and their potential impact on plant–insect interactions [35–38].

Among the known endophytic volatiles, sesquiterpenes have gained much attention in recent years as they can play an important role in plant–plant, plant–microbe, and microbe–microbe interactions [39–40]. Weikl et al., for instance, analyzed the volatile emission of *Alternaria alternata* and *Fusarium oxysporum* in culture and showed that both species are able to produce sesquiterpenes like (*E*)-β-farnesene (**2**), α- and β-chamigrene (**4**), and germacrene D [41]. In general, terpenes are derived from the five-carbon intermediates dimethylallyl diphosphate (DMAPP) and isopentenyl diphosphate (IPP), which are both produced by the mevalonate pathway in fungi [42]. The condensation of DMAPP with varying numbers of IPP residues results in products of various chain lengths: geranyl diphosphate (GPP, C10), farnesyl diphosphate (FPP, C15), and geranylgeranyl diphosphate (GGPP, C20). Terpene synthases (TPS) then convert the precursors GPP, FPP, and GGPP into the different terpene skeletons [42–44]. However, our knowledge on terpene synthases of endophytic fungi is scarce, specifically in comparison to the vast knowledge on these enzymes in plants and bacteria [44–45].

Typical monoterpenes like limonene and linalool (**5**), sesquiterpenes like α-farnesene, chamigrene (**4**), aromatic alcohols like 2-phenylethanol (**3**), and aliphatic alcohols like 3-methyl-1-butanol (**6**) are also found in the headspace of endophytic fungi grown in culture [46–52]. Those studies have shown that volatile blends produced by some endophytic fungi qualitatively overlap with the VOC bouquets produced by numerous plant species [53–56] including black poplar (*Populus nigra*) [57–59]. Thus, the question arises whether endophytes found in plants contribute significantly to the overall plant volatile blend by expression of their own *TPS* genes and how these fungal volatiles influence plant–insect interactions. Identification of fungal *TPS* genes is a useful tool to assess the impact of fungal terpene emission on plant volatile composition and on plant–insect interactions.

In this study, we isolated and identified endophytic fungi from leaves of a natural population of mature black poplar trees. From these fungi, we qualitatively investigated the volatiles emitted in culture and compared the blend with that emitted from black poplar trees. In addition, we used transcriptome analysis and heterologous expression to identify and characterize terpene synthases in one of the endophyte species isolated. These fungal TPSs may contribute to the volatile blend of black poplar foliage and the compounds emitted may play a role in poplar plant–insect interactions.

## Results

### Endophytic fungi isolated from old-growth black poplar trees

We identified 12 endophyte species from nine different genera by sequencing the internal transcribed spacer (ITS) region of the nuclear ribosomal RNA cistron. Two species were identified from the genus *Alternaria*, three from *Didymella*, two from *Aureobasidium*, and one each from *Arthrinium*, *Cladosporium*, *Fusarium*, *Sordaria*, and *Stemphylium* (Table 1). One unidentified species was also included in the volatile analysis. All the identified fungi belong to the Ascomycota, the largest fungal phylum.

**Table 1:** Fungal endophytes identified from leaves of mature black poplar (*Populus nigra*) trees.^a^

Species	Family	Best hit and accession number	Identity (%)

*Alternaria infectoria*	Pleosporaceae	*Alternaria infectoria* KX394561.1	100
*Alternaria* sp. 1	Pleosporaceae	*Alternaria* sp. KY788045.1	99
*Stemphylium* sp.	Pleosporaceae	*Stemphylium* sp. KX400960.1	99
*Aureobasidium* sp. 1	Dothioraceae	*Aureobasidium pullulans* KX869960.1	100
*Aureobasidium* sp. 2	Dothioraceae	*Aureobasidium pullulans* KT352844.1	97
*Didymella glomerata*	Didymellaceae	*Didymella glomerata* KY788126.1	99
*Didymella* sp. 1	Didymellaceae	*Didymella glomerata* KY788126.1	100
*Didymella* sp. 2	Didymellaceae	*Didymella glomerata* KY794938.1	100
*Cladosporium* sp.	Cladosporiaceae	*Cladosporium subcinereum* NR_148193.1	100
*Fusarium* sp.	Nectriaceae	*Fusarium armeniacum* KF944456.1	100
*Sordaria* sp.	Sordariaceae	*Sordaria fimicola* KX986578.1	100
*Arthrinium* sp.	Apiosporaceae	*Arthrinium sacchari* KY782634.1	100
unidentified species			

^a^Endophytes were isolated from leaves after surface sterilization (*n* = 10 tree genotypes)*.* 12 out of 13 isolated endophytes were classified to the genus level via sequencing of the internal transcribed spacer (ITS) region of the nuclear ribosomal cistron (with primers ITS1F/ ITS4). The sequences obtained were compared to the NCBI sequence database (Supporting Information File 1, Table S1). Isolated fungi with multiple 99–100% identity hits on several species within the same genus were identified only to the genus level, but we still list the single best hit in the table.

### Endophytic fungi emit typical plant VOCs

**Figure 1:**
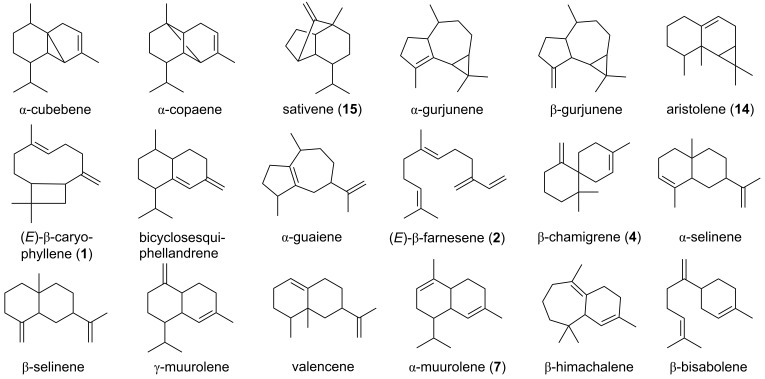
Chemical structures of sesquiterpenes emitted from endophytic fungi (Table 2) isolated from black poplar leaves.

Altogether, we detected 77 volatile compounds in the headspaces of the 13 different endophytic species grown on agar medium. With 34 different compounds, the unidentified fungus was the endophyte emitting the most complex volatile blend. In contrast, in the headspace of both *Stemphylium* sp. and *Cladosporium* sp., only two volatile compounds were detected (Table 2). All endophytic fungi, except *Cladosporium* sp., produced aliphatic or aromatic alcohols like 2-methyl-1-propanol (**7**), 3-methyl-1-butanol (**6**) or 2-phenylethanol (**3**). Of 77 detected volatile compounds, 50 compounds are sesquiterpenes. Furthermore, seven out of 13 fungi produced sesquiterpenes. In general, the analyzed endophytic fungi have a species-specific volatile bouquet, and none of the endophytic species shared the same combination of volatile compounds. We had previously detected a number of these fungal volatiles in our volatile analyses of poplar leaves, including two alcohols 3-methyl-1-butanol (**6**) and 2-phenylethanol (**3**) and the two sesquiterpenes (*E*)-β-caryophyllene (**1**) and α-muurolene (**8**) (Table 2, Figure 1) [7,9,57–59].

**Table 2:** Volatiles emitted from endophytic fungi growing in culture on potato dextrose agar (PDA).^a^

			Endophyte species												
	
Volatiles class	Volatile organic compound	Kovats' RI	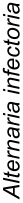	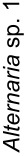	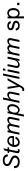	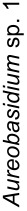	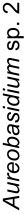	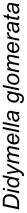	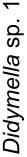	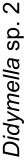	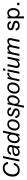	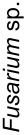	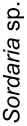	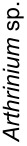	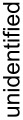

aliphatic alcohol	ethanol (**17**)^b^			**X**		**X**	**X**	**X**	**X**	**X**		**X**	**X**	**X**	**X**
aliphatic ketone	2-butanone	598						**X**		**X**					
aliphatic ester	ethyl acetate	611				**X**									
aliphatic alcohol	2-methyl-1-propanol (**7**)	623	**X**	**X**	**X**	**X**	**X**	**X**	**X**	**X**				**X**	
–	unknown 1	658													**X**
aliphatic alcohol	3-hydroxy-2-butanone	710				**X**	**X**	**X**	**X**	**X**			**X**		**X**
aliphatic alcohol	**3-methyl-1-butanol (6)^b^**	730			**X**	**X**	**X**	**X**	**X**	**X**			**X**	**X**	**X**
aliphatic alcohol	2-methyl-1-butanol	732	**X**	**X**											
–	unknown 2	776													**X**
aliphatic ester	3-methylbutyl acetate	881				**X**								**X**	
aromatic hydrocarbon	ethenylbenzene	891									**X**				
–	unknown 3	907				**X**									
–	unknown 4	1044												**X**	
–	unknown 5	1054	**X**												
aromatic alcohol	**2-phenylethanol (3)^b^**	1115				**X**	**X**							**X**	
sesquiterpene	unknown 6	1335	**X**	**X**											
sesquiterpene	unknown 7	1343	**X**	**X**											**X**
sesquiterpene	α-cubebene	1355													**X**
–	unknown 8	1356						**X**							
–	unknown 9	1361						**X**							
–	unknown 10	1369						**X**							
sesquiterpene	unknown 11	1372													**X**
sesquiterpene	α-copaene	1381													**X**
–	unknown 12	1391													**X**
sesquiterpene	unknown 13	1395													**X**
sesquiterpene	sativene (**16**)	1401													**X**
sesquiterpene	α-gurjunene	1415	**X**	**X**											
sesquiterpene	unknown 14	1416										**X**			
sesquiterpene	unknown 15	1419													**X**
sesquiterpene	unknown 16	1420										**X**			
sesquiterpene	unknown 17	1423													**X**
sesquiterpene	aristolene (**15**)	1424	**X**	**X**											
sesquiterpene	**(*E*)-β-caryophyllene (1)^b^**	1425									**X**				
sesquiterpene	unknown 18	1426													**X**
sesquiterpene	unknown 19	1433	**X**	**X**											
sesquiterpene	unknown 20	1433						**X**							**X**
sesquiterpene	bicyclosesquiphellandrene	1436										**X**			
sesquiterpene	β-gurjunene^c^	1437	**X**	**X**											
sesquiterpene	unknown 21	1438													**X**
sesquiterpene	unknown 22	1440										**X**			
sesquiterpene	unknown 23	1442	**X**	**X**											
sesquiterpene	α-guaiene^c^	1447	**X**	**X**											
sesquiterpene	unknown 24	1448										**X**			
sesquiterpene	unknown 25	1453										**X**			
sesquiterpene	unknown 26	1454													**X**
sesquiterpene	(*E*)-β-farnesene (**2**)^c^	1456										**X**			
sesquiterpene	unknown 27	1462													**X**
sesquiterpene	unknown 28	1467													**X**
sesquiterpene	unknown 29	1469										**X**			
sesquiterpene	unknown 30	1472	**X**	**X**											
sesquiterpene	β-chamigrene^c^	1474										**X**			
sesquiterpene	unknown 31	1475	**X**												
–	unknown 32	1475													**X**
sesquiterpene	α-selinene^c^	1477	**X**	**X**											
sesquiterpene	γ-muurolene	1478													**X**
sesquiterpene	unknown 33	1483													**X**
sesquiterpene	unknown 34	1486	**X**	**X**											
sesquiterpene	β-selinene	1488	**X**	**X**											
sesquiterpene	unknown 35	1489													**X**
sesquiterpene	valencene^b^	1494	**X**	**X**											
sesquiterpene	unknown 36	1498	**X**	**X**											
sesquiterpene	**α-muurolene (8)**	1500													**X**
sesquiterpene	β-himachalene	1502										**X**			
sesquiterpene	β-bisabolene	1508										**X**			**X**
–	unknown 37	1525										**X**			
sesquiterpene	unknown 38^d^	1525													**X**
sesquiterpene	unknown 39	1533										**X**			
sesquiterpene	unknown 40	1544										**X**			
oxygenated ST	unknown 41	1549													**X**
–	unknown 42	1553												**X**	
sesquiterpene	unknown 43	1564													**X**
–	unknown 44	1584													**X**
oxygenated ST	unknown 45	1609	**X**	**X**											
–	unknown 46	1629													**X**
–	unknown 47	1650													**X**
–	unknown 48	1656													**X**
–	unknown 49	1702													**X**

^a^Volatiles were verified with authentic standards, or identified by comparing their mass spectra with reference spectra from databases (Wiley, NIST). Kovats’ retention indices (RI) were calculated and compared to databases. Volatile organic compounds collected as background from fungal-free PDA plates were removed from the final dataset. Volatiles released from both the endophytic fungi and black poplar, as listed in previous reports [57–58], are depicted in bold. ^b^Verified with authentic standards, otherwise verified with calculated Kovat’s indices compared with Pubchem [60] or ^c^NIST [61] library. ^d^Kovat’s indices and mass spectra suggest strongly resemblance to β-or γ-cadinene.

### *Cladosporium* sp. contains two sesquiterpene synthases that produce typical poplar volatile compounds in in vitro assays

The poplar fungal endophyte *Cladosporium* sp. emitted (*E*)-β-caryophyllene (**1**) in culture (Table 2, Figure 1). As this sesquiterpene is also a characteristic VOC in the constitutive and herbivore-induced blends of black poplar [57–59], we wanted to identify and characterize the responsible fungal terpene synthase, as this enzyme could contribute to the overall (*E*)-β-caryophyllene emission from the tree.

To identify terpene synthase genes potentially involved in volatile terpene formation in *Cladosporium,* we sequenced the transcriptome and performed a de novo assembly of the obtained reads. A TBLASTN analysis with *Aspergillus terreus* aristolochene synthase (pdb 20A6) as query and the de novo assembly as template revealed two genes with high similarity to other fungal *TPS* genes. The genes were designated *CxTPS1* and *CxTPS2*. For functional characterization, the complete open reading frames of *CxTPS1* and *CxTPS2* were amplified from cDNA, cloned, and heterologously expressed in *Escherichia coli*. To determine mono-, sesqui-, and diterpene-forming activity, the bacterial raw protein extracts were assayed with the substrates GPP, FPP, and GGPP, each in the presence of the co-substrate magnesium chloride.

**Figure 2:**
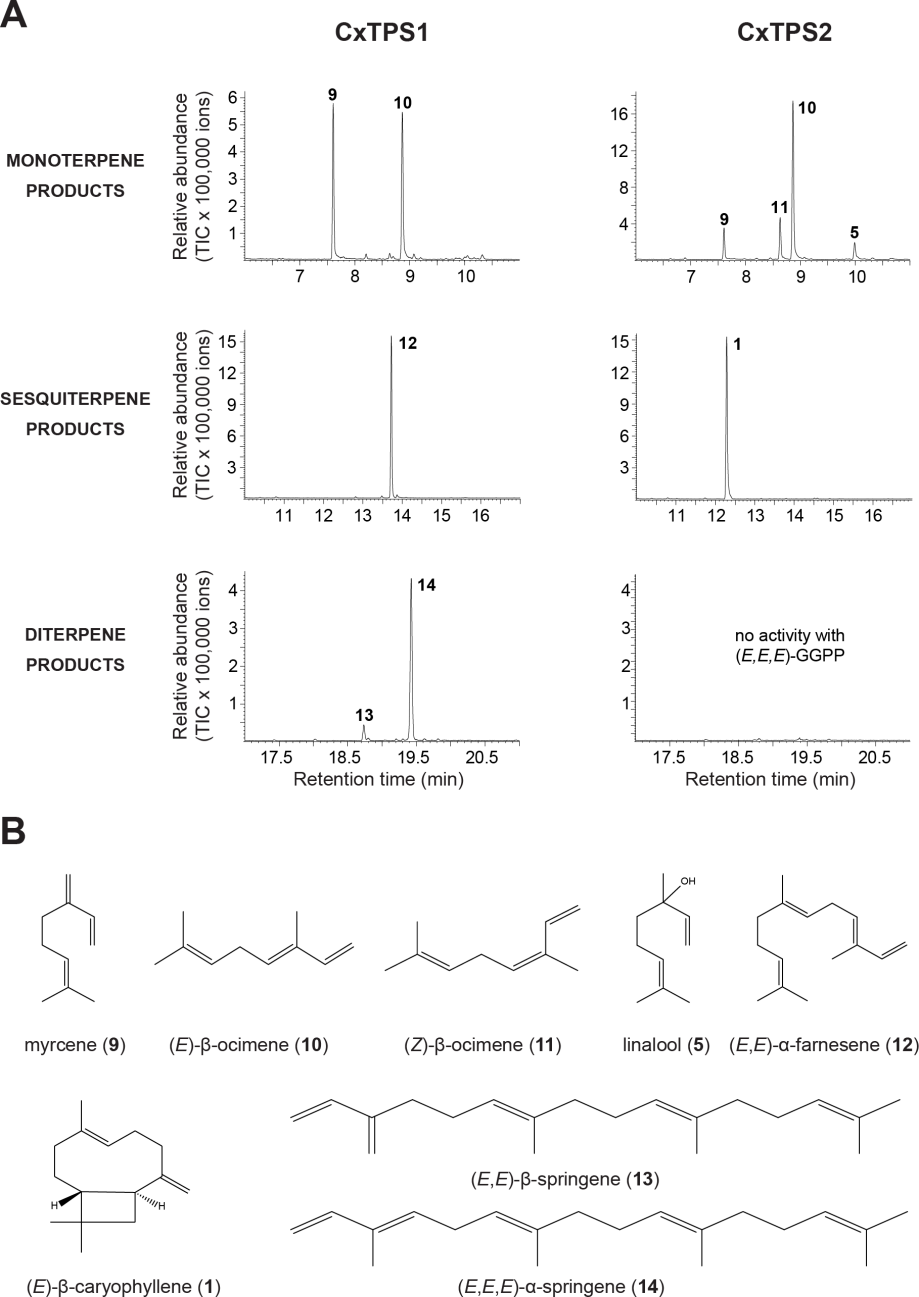
Terpene synthase activity of CxTPS1 and CxTPS2. A) Genes were heterologously expressed in *Escherichia coli* and partially purified proteins were assayed with GPP, (*E*,*E*)-FPP, or (*E*,*E*,*E*)-GGPP as substrates in the presence of 10 mM MgCl_2_. Enzyme products were extracted from the assays with hexane and analyzed using gas chromatography–mass spectrometry. Myrcene (**9**); (*E*)-β-ocimene (**10**); (*Z*)-β-ocimene (**11**); linalool (**5**); (*E*,*E*)-α-farnesene (**12**); (*E*)-β-caryophyllene (**1**); (*E*,*E*)-β-springene (**13**); (*E*,*E*,*E*)-α-springene (**14**). B) Structures of the enzyme products of CxTPS1 and CxTPS2, including (*E*)-β-caryophyllene (**1**) which was the only terpene detected from *Cladosporium* sp. cultures.

Both protein extracts containing the respective enzymes accepted the substrate GPP and produced monoterpenes (Figure 2). CxTPS1 produced myrcene (**9**) and (*E*)-β-ocimene (**10**) in similar amounts. CxTPS2 produced (*E*)-β-ocimene (**10**) as the major product and minor amounts of myrcene (**9**), (*Z*)-β-ocimene (**11**), and linalool (**5**) (Figure 2). Only one sesquiterpene product was formed by each TPS: CxTPS1 produced (*E*,*E*)-α-farnesene (**12**) and CxTPS2 produced (*E*)-β-caryophyllene (**1**). With GGPP, no enzyme activity was recorded for CxTPS2, while CxTPS1 converted this substrate to (*E,E*)-β-springene (**13**) as the minor compound and major amounts of (*E,E,E*)-α-springene (**14**) (Figure 2).

### Two terpene synthases from *Cladosporium* sp. are not closely related to each other

To investigate the phylogenetic relationships of CxTPS1 and CxTPS2 from *Cladosporium* sp. to other known terpene synthases from plant-associated Ascomycota that exhibit a pathogenic, endophytic or saprophytic lifestyle, we performed multiple sequence alignments and a subsequent dendrogram analysis.

**Figure 3:**
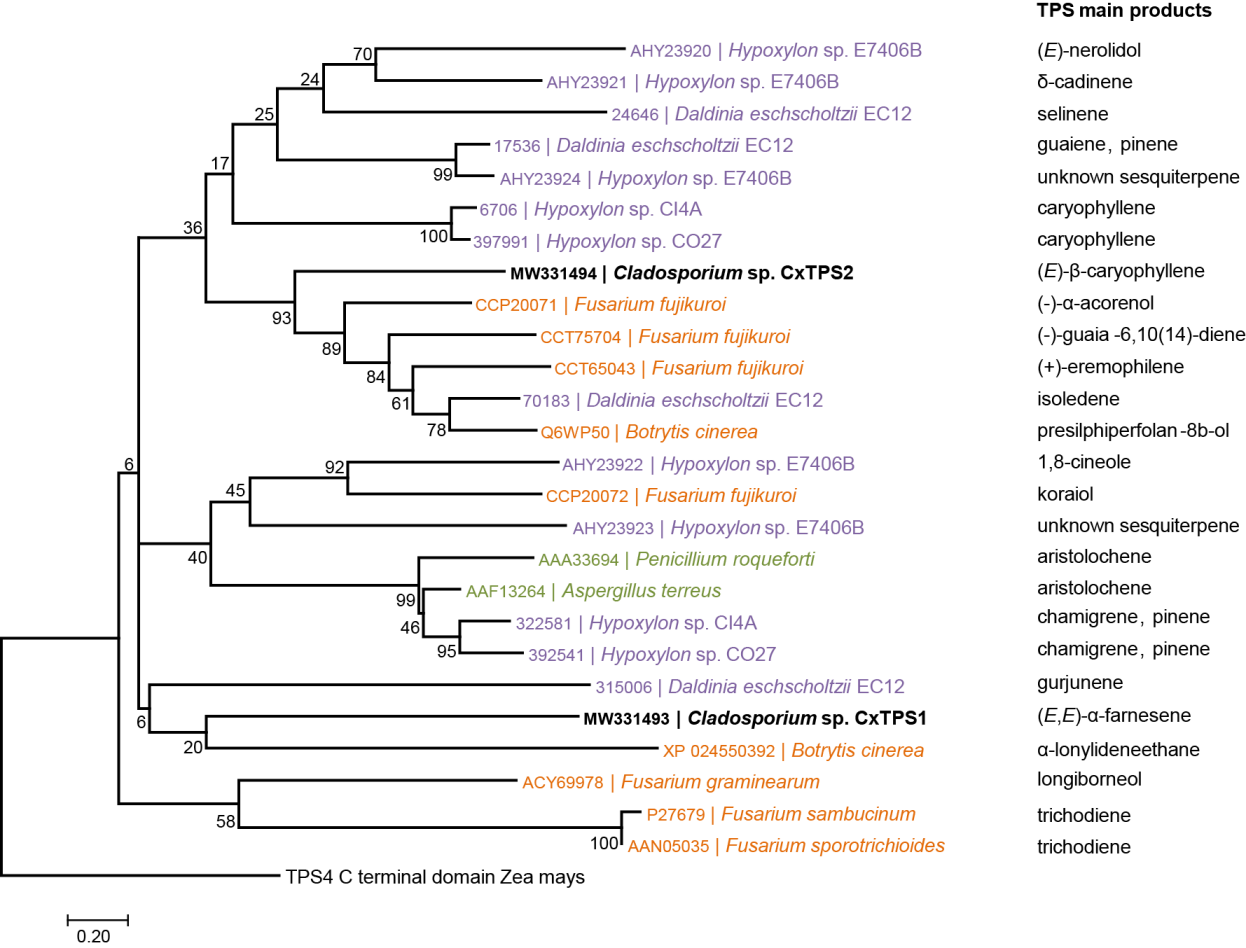
Dendrogram analysis (rooted tree) of CxTPS1 and CxTPS2 (bold) from *Cladosporium* sp. and characterized TPS proteins and their main products from other plant-associated Ascomycota. The tree was inferred using the Maximum Likelihood method based on the Poisson correction model and *n* = 1000 replicates for bootstrapping. Bootstrap values are shown next to each node. The tree is drawn to scale, with branch lengths measured in the number of amino acid substitutions per site. The alpha-domain of maize TPS4 [62] was chosen as an outgroup. TPS proteins from different Ascomycota are highlighted according to their different lifestyle: endophytic (purple), pathogenic (orange) and saprophytic (green).

According to the tree shown in Figure 3, CxTPS2 and CxTPS1 are not closely related to each other. While CxTPS2 forms a clade with sesquiterpene synthases of four pathogenic fungi and one endophyte, CxTPS1 is loosely related to a gene of the pathogenic fungus *Botrytis cinerea*. Further, CxTPS2, which produces (*E*)-β-caryophyllene (**1**), is more closely related to other sesquiterpene synthases from pathogens than to the caryophyllene synthases from the two endophytes *Hypoxylon* sp. CI4A and *Hypoxylon* sp. CO27.

## Discussion

We were able to identify 12 different endophytic fungi from leaves of mature black poplar trees with a culture-dependent method and analyzed their volatile blends when growing on potato dextrose agar. Most of the tested fungi produced various aliphatic or aromatic alcohols, which are commonly produced by endophytic fungi and are known to act as antimicrobial agents (Table 2) [63]. Sesquiterpenes make up the largest proportion of fungus-produced terpenoids [64] and in our study we also detected several sesquiterpenes, e.g., (*E*)-β-caryophyllene (**1**), β-chamigrene (**4**), aristolene (**15**), sativene (**16**), and α-muurolene (**8**). However, monoterpenes were completely absent from the volatile bouquets of the endophytic species in our study. Weikl et al. who compared the volatiles released from *Alternaria alternata* and *Fusarium oxysporum* also did not detect any monoterpenes [41]. However, other studies on *Phomopsis* sp., *Cladosporium cladosporioides*, and *Hypoxylon anthochroum* showed that these endophytic fungi are able to produce monoterpenes like sabinene, α-pinene and 1,8-cineole, respectively [51,65–66]. In general, fungal volatile profiles are very species-specific [67], which also holds true for the species tested in our study (Table 2). However, the differences in the literature may arise from the use of different strains, volatile collection methods or variation in age, growth medium and environmental conditions, such as moisture, pH, temperature, and nutrient levels, or co-cultivation [27,41,67–68]. In our study, we measured the volatile profiles of endophytes cultivated on PDA medium at 28 °C in the dark. These profiles may differ from those released by endophytes growing under natural conditions in poplar leaves, in the possible presence of competing microbes.

While our knowledge about the volatile profiles of endophytic fungi has increased in recent years, only little is known about endophyte terpene synthases that may catalyze volatile terpene formation [44–45]. For the endophytic fungus *Cladosporium* sp., we identified and characterized two TPS, CxTPS1 and CxTPS2 (Figure 2). CxTPS1 was a multifunctional enzyme in vitro and produced the monoterpenes myrcene (**9**) and (*E*)-β-ocimene (**10**) from GPP, the sesquiterpene (*E*,*E*)-α-farnesene (**12**) from FPP, and the diterpenes (*E*,*E*)-β-springene (**13**) and (*E*,*E*,*E*)-α-springene (**14**) from GGPP. CxTPS2, in contrast, showed a narrower substrate specificity and converted GPP to myrcene (**9**), (*E*)-β-ocimene (**10**), (*Z*)-β-ocimene (**11**), and linalool (**5**) and FPP to (*E*)-β-caryophyllene (**1**). In a previous work on fungal terpene synthases, Hohn and Vanmiddlesworth found a narrow substrate specificity for the trichodiene synthase from *Fusarium sporotrichioides*, where only the sesquiterpene trichodiene was detected with FPP, while other substrates were not accepted [69]. In contrast, bi-functionality was also observed for the pinene and guaiene synthase from *Daldinia eschscholzii* EC12 and the pinene and guaiene synthase from *Hypoxylon* sp. EC28 (Figure 3) [45]. The multifunctionality of CxTPS1 and CxTPS2 was only observed when the fungal TPS was expressed heterologously in *E. coli* and assayed in vitro whereas the fungus itself only emitted (*E*)-β-caryophyllene (**1**) when growing on agar medium. Thus, we speculate that GPP, the substrate for monoterpene production, is not available in *Cladosporium* sp. In contrast, the emission of the monoterpene α-pinene has been reported for *Cladosporium cladosporioides* CL-1 [66]. Interestingly, we could not detect the emission of (*E*,*E*)-α-farnesene (**12**), a product of the in vitro assay of CxTPS1 in our fungal cultures, although the fungus must have the ability to produce the substrate FPP in sufficient quantity as it also produces the sesquiterpene (*E*)-β-caryophyllene (**1**). It might be that *CxTPS1* is not expressed in the fungus under our culture conditions or that (*E*,*E*)-α-farnesene (12) is further metabolized. To our knowledge, (*E*,*E*)-α-farnesene (**12**) has never been detected so far from any Cladosporium species.

To test whether there is a relationship between fungal lifestyle and their terpene synthases, we compared sequences of the terpene synthases CxTPS1 and CxTPS2 from *Cladosporium* sp. with the sequences of other known terpene synthases from plant-associated Ascomycota exhibiting a pathogenic, endophytic or saprophytic lifestyle. One clade was indeed evident that contained only terpene synthases from endophytes. However, a close relationship between fungal lifestyle and their terpene synthase sequences is not observable, since different terpene synthases from the same fungal species clustered together with terpene synthases from pathogens and/or endophytes (Figure 3). CxTPS2 forms a clade with sesquiterpene synthases from four pathogenic fungi and one endophyte, while CxTPS1 is loosely related to sequences of the pathogen *Botrytis cinerea* (Figure 3)*.* We speculate that TPS from fungi that share the same lifestyle are not clustered together because some endophytes switch from being asymptomatic leaf inhabiting fungi to becoming either latent pathogens or saprophytes [21,24,70–73]. Furthermore, it is hypothesized that endophytes may have evolved directly from pathogens, since both must defeat plant protective barriers [38,74]. Nevertheless, the bootstrap values in the dendrogram are generally too low to make a clear statement about the relationship between terpene synthases and fungal lifestyle, and more work on this question is needed [63,75].

The volatiles found to be emitted from black poplar endophytic fungi in this study could have important biological activities. For instance, ethanol (**17**) and 2-phenylethanol (**3**) are known to have antifungal and phytotoxic activity and so could help the endophyte to defend its niche within the plant against other endophytic competitors [63]. The other endophyte VOCs could promote plant growth (e.g., 2-methyl-1-propanol (**7**) [76], (*E*)-β-caryophyllene (**1**) [77], and sativene (**16**) [66]), induce plant immunity (e.g., (*E*)-β-caryophyllene (**1**) [77]), and increase photosynthetic capacity (e.g., 2-methyl-1-propanol (**7**) [78]) (Table 2) [63]. Some of the analyzed compounds are also known to play a crucial role in plant–insect interactions, where they are involved in direct and indirect plant defenses or in attracting herbivorous insects. For example, (*E*)-β-caryophyllene (**1**) emitted by *Cladosporium* sp. (Table 2) is known to act as a signal cue for the planthopper *Sogatella furcifera* [15], while this compound also attracts nematodes that feed on attacking insect herbivores [79]. Nearly all of the endophytic fungi isolated in this study were able to produce at least some volatiles known from the literature to mediate plant–insect interactions.

Of the 13 endophytes studied, 11 of them release volatiles previously reported from black poplar foliage (Table 2) [57–59]. These compounds include the alcohols 3-methyl-1-butanol (**6**) and 2-phenylethanol (**3**) and the sesquiterpenes (*E*)-β-caryophyllene (**1**), and α-muurolene (**8**) (Table 2). This raises the question of whether endophytic fungi contribute to the overall plant volatile bouquet by producing the above-mentioned volatiles. If so, this would directly affect our interpretation of certain plant-fungus and plant–insect interactions [34,37–38]. Recently, it has been shown that the pathogenic rust fungus (*Melampsora larici-populina*) alters the volatile blend of black poplar trees by contributing 1-octen-3-ol and 3-octanone, which attract caterpillars of the generalist herbivore *Lymantria dispar* [57]. Jallow et al. showed that an endophytic fungus (*Acremonium strictum*) alters the volatile composition of the tomato plant *Solanum lycopersicum* and attracts *Helicoverpa armigera* moth for oviposition [80]. The endophytic fungus *Beauveria bassiana* also increased the emission of some terpenes from tomato plants resulting in a stronger defense response against the beet armyworm (*Spodoptera exigua*) [81]. In these cases, it is not known whether the increased terpene emission results from biosynthesis by the plant or the fungus. Future work should include measurements of plant and fungal TPS expression to determine the origin of these compounds. For this, identification of TPS genes in both plants and their fungal partners is crucial.

## Conclusion

We showed that endophytic fungi isolated from mature black poplar trees emitted species-specific volatile blends. Almost all the endophytes here produced short-chain aliphatic alcohols that are known to have antifungal and phytotoxic effects and may be produced to compete with other microbial species. Several also produce diverse mixtures of sesquiterpenes. Interestingly, several VOCs emitted from the endophytes were earlier reported to be emitted by black poplar. We characterized two terpene synthases from one of the endophytic fungi to lay the groundwork for comparing the biosynthesis of plant vs fungal volatiles. More knowledge about the formation of these compounds could contribute to the greater understanding of their roles in plant–insect, plant–plant and plant–microbe interactions.

## Experimental

### Endophyte isolation from plant material

Endophytes were isolated from leaves of mature black poplar (*Populus nigra*) trees growing in a natural population in a floodplain forest in northeastern Germany (52°34’1’’N, 14°38’3’’E). The trees were around 25 m in height and approximately 70 years old. Five branches in the lower canopy (1–7 m) from each of the 10 tree genotypes were collected and from each branch, five leaves were randomly harvested. Those leaves did not show any symptoms of pathogen infection. A culture-dependent method was used to isolate endophytic fungi growing within the leaf blades. Under a clean bench, the leaves were surface sterilized (0.5% NaOCl for 2 min, followed by 70% of EtOH for 2 min) and rinsed three times by immersion in sterile distilled water. Then, four pieces (approximately 7 × 7 mm) of one leaf blade were placed equidistantly on potato dextrose agar (PDA; Sigma-Aldrich). Water from the last washing step was coated on PDA medium to test whether the surface of the leaves had been adequately sterilized. Petri dishes were sealed with Parafilm and incubated in the dark at 25 °C. Plates were inspected daily and morphologically distinct colonies were brought into pure culture on PDA medium using the same culturing conditions as above. Fresh mycelium was harvested from pure cultures for molecular identification of the morphospecies.

### Molecular identification of endophytic fungi

DNA was extracted from fresh mycelium (approximately 5 cm in diameter) growing on PDA. The mycelium was flash frozen in liquid nitrogen and ground using plastic pestles in 1.5 mL Eppendorf tubes. After homogenization of the mycelium, 500 µL extraction buffer (100 mM Tris HCl, pH 8; 10 mM EDTA, pH 8; 2% w/v SDS) and 100 µL proteinase K (Sigma) were added and the mixture was incubated for 1 h at 60 °C. For separation of polysaccharides, 180 µL 5 M NaCl and 80 µL 10% CTAB were added and the mixture incubated further for 10 min at 65 °C.

To extract nucleic acids by phase separation, 860 µL chloroform/isoamyl alcohol (24:1) was added and incubated on ice for 30 min. The samples were centrifuged for 10 min (15,000 rpm), and the upper, aqueous phase was then transferred to a new tube and DNA was precipitated in 395 µL of 100% isopropanol (−20 °C). After centrifugation (4 °C, 20 min, 15,000 rpm) the pellet was washed with 750 µL 70% ethanol, centrifuged at 15,000 rpm (10 min), dried, and finally dissolved in 50 µL Milli-Q water (pH 6). DNA concentration and purity were determined with a NanoDrop 2000c spectrophotometer (Peqlab Biotechnology AG, Erlangen, Germany).

The primer pair ITS1F and ITS4 (Supporting Information File 1, Table S2) was used to amplify the highly conserved internal transcribed spacer region of the fungal rRNA cistron [82–83]. The reaction mix for DNA amplification (50 µL/tube) contained 2.5 µL of each primer (Sigma), 0.5 µL GoTaqX^®^ Polymerase (Promega, Madison, WI, USA), 10 µL of GoTaqX^®^ Reaction Buffer (Promega) and 1 µL 10 mM dNTPs (Thermo Fisher Scientific). The template volume was adjusted to a final DNA concentration of approximately 500 ng/mL. Ultrapure water (Milli-Q^®^ Synthesis A10) was added up to a final volume of 50 µL. PCR was performed in a gradient thermal cycler (Whatman Biometra 96T) using the following program: initiation and activation of polymerase (95 °C/5 min); followed by 35 cycles of denaturation (95 °C/30 s), annealing (65 °C/30 s) and elongation (72 °C/90 s) and a single, final elongation step (72 °C/10 min).

For gel electrophoresis, 4 µL PCR product was mixed with one drop loading dye (0.3 mL 30% glycerol and 2.5 mg bromphenol blue/mL) and applied to an 1% agarose gel (1 g agarose/100 mL 0.5% TBE; 5 µL Midori Green). A 1 kb DNA ladder (Gene Ruler, Thermo Fisher Scientific) was applied to determine the fragment size of the products. Electrophoresis was performed in 0.5% TBE buffer (Thermo Fisher Scientific) for 30 min at 135 V (150 mA). The PCR products were purified with a QIAquick PCR purification kit (Qiagen, Hilden, Germany) following the manufacturer’s instructions. Purified PCR products were sequenced using the Sanger method on a ABI Prism^®^ Gen-Analysator 3130xl (Applied Biosystems, Weiterstadt, Germany). The obtained sequences were aligned using Geneious 6.0.5 [84] and compared to the NCBI sequence database [85] (Supporting Information File 1, Table S1). In case of isolates with multiple 99–100 % identity hits on several species within the same genus, we identified these only to the genus level, but still list the single best hit and its accession number (Table 1, Supporting Information File 1, Table S1).

### Static headspace volatile collection from cultures and analysis

VOCs were collected from endophytes that had grown on PDA medium (25 mL) in an incubator (dark/28 °C) until the mycelium reached a diameter of 5 cm (± 0.5 cm). For each fungal species, seven replicates were used with fungus-free petri dishes with PDA medium used as blanks. Volatiles were trapped for 1 h by using four polydimethylsiloxane (PDMS) tubes. To prevent PDMS tubes from touching the mycelium, the tubes were placed with watchmaker forceps on loops of stainless steel wire that were kept at a distance of approximately 5 mm from the mycelium. PDMS tubes were prepared following the method described in Kallenbach et al. [86]. The experiment was performed under a clean bench at room temperature. After volatile collection PDMS tubes were immediately removed from the wire and stored in glass vials at −20 °C until further analysis.

Volatiles trapped on PDMS tubes were analyzed by GC–MS (GCMS-QP2010 Ultra, Shimadzu, Duisburg, Germany) coupled to a thermal desorption unit (TD-20, Shimadzu, Duisburg, Germany). A single PDMS tube from each replicate was placed in a glass tube (Supelco; Sigma-Aldrich). Desorption was achieved by a He flow (60 mL min^−1^) at 200 °C for 8 min in the glass tube and the analytes were trapped on a Tenax^®^ (Buchem BV, Apeldoorn, Netherlands) adsorbent trap at −17 °C. The trap was then heated to 230 °C, and the analytes injected onto the GC column (Rtx^®^-5MScolumn with 30 m × 0.25 mm × 0.25 μm (Restek GmbH, Bad Homburg, Germany)). The gas chromatograph was operated at a column flow rate of 1.5 mL/min (He), split injection (split ratio: 5). The oven was set to 45 °C, held for 3 min, increased to 250 °C with a gradient of 6 °C/min and subsequently increased to 300 °C at 100 °C/ min with a 3 min hold. Electron impact (EI) mass spectra were recorded at 70 eV in scan mode from 43 to 350 *m*/*z* at a scan speed of 1111 Da/s (interface temperature, 250 °C; source temperature, 230 °C). Fungal volatiles were identified by comparing their mass spectra with those of authentic standards or reference spectra from databases (Wiley, Version 8, National Institute of Standards and Technology (NIST, Version 11)) using GCMS SOLUTION v.4.20 (Shimadzu). In addition, non-isothermal Kovats retention indices were calculated, based on chromatographic retention times of a saturated alkane mixture (C_7_–C_40;_ Sigma-Aldrich, Taufkirchen, Germany) [87]. The calculated Kovats retention indices were compared with indices published in Pubchem [60] or NIST [61] from the same or a similar type of GC column. Differences between calculated retention index and literature data were within ±5 points. Identified volatiles with a similarity hit above 90% and that were present in five out of seven replicates were included in this study, whereas VOCs which were also collected by blanks were removed from the final dataset. A representative total ion chromatogram for each fungus is shown in Supporting Information File 1, Figure S1. Mass spectra of unknown compounds are shown in Supporting Information File 1, Figure S2.

### Fungal RNA extraction, reverse transcription, and sequencing

Total RNA was isolated from fresh mycelium (approximately 5 cm in diameter) growing on PDA using the RNeasy^®^ Plant Mini Kit (Qiagen) according to the manufacturer’s instructions. The RNA concentration was assessed using a spectrophotometer (NanoDrop 2000c, Thermo Fisher Scientific). RNA was treated with DNase I (Thermo Fisher Scientific) prior to cDNA synthesis. Single-stranded cDNA was prepared from 1 µg of DNase-treated RNA using SuperScriptTM III reverse transcriptase and oligo (dT12-18) primers (Invitrogen, Carlsbad, CA, USA).

For transcriptome sequencing, total RNA was extracted from fungal material as described above, a TruSeq RNA-compatible library was prepared, and PolyA enrichment was performed before sequencing on an IlluminaHiSeq 3000 sequencer (Max Planck Genome Centre, Cologne, Germany) with 25 Mio reads, 150 base pairs, paired end. Trimming of the obtained Illumina reads and de novo assembly were both performed with the program CLC Genomics Workbench (Qiagen Bioinformatics) using default parameters or parameters specified as follows: bubble size, 100; automatic word size; minimum contig length, 600. A BUSCO analysis (Supporting Information File 1, Figure S3) was performed to validate the completeness of the transcriptome.

### Identification and heterologous expression of terpene synthase genes

To identify putative terpene synthases, a TBLASTN analysis with *Aspergillus terreus* aristolochene synthase (pdb 20A6) as query and the de novo transcriptome of *Cladosporium* sp. as a template was performed using the software BioEdit 7.0.9.0 [88]. Two terpene synthase-like sequences were found and designated as *CxTPS1* and *CxTPS2,* respectively. The complete open reading frames of *CxTPS1* and *CxTPS2* were amplified from cDNA using the primers shown in Supporting Information File 1 (Table S2) and cloned into pET100/D-TOPO vector (Thermo Fisher Scientific). The *E. coli* strain BL21 Star™ (DE3) (Thermo Fisher Scientific) was used for heterologous expression. The culture was grown at 37 °C, induced at an OD_600_ = 0.6 with 1 mM IPTG, and subsequently placed at 18 °C and grown for another 20 hours. The cells were collected by centrifugation and disrupted by a 4 × 20 s treatment with a sonicator (Bandelin UW2070, Berlin, Germany) in chilled extraction buffer (10 mM Tris-HCl, pH 7.5, 1 mM dithiothreitol, 10% (v/v) glycerol). Cell fragments were removed by centrifugation at 14,000*g* and the supernatant was further processed via an Illustra NAP-5 gravity flow desalting column (GE Healthcare, Chicago, IL, USA) and eluted in extraction buffer.

Enzyme assays were performed in a Teflon-sealed, screw-capped 1 mL GC glass vial containing 50 μL of the heterologously expressed protein and 50 µL assay buffer containing 50 μM substrate (GPP, (*E,E*)-FPP, or (*E,E,E*)-GGPP) and 20 mM MgCl_2_. Assays were overlaid with 100 µL hexane and incubated for 60 minutes at 30 °C. One microliter of the hexane phase was injected into the GC–MS machine and the analysis was conducted using an Agilent 6890 Series gas chromatograph coupled to an Agilent 5973 quadrupole mass selective detector (interface temp, 270 °C; quadrupole temp, 150 °C; source temp, 230 °C; electron energy, 70 eV). Chromatographic separation was achieved with an initial oven temperature of 45 °C held for 2 min, which was then increased to 180 °C with a gradient of 6 °C min^−1^, and then further increased to 300 °C with a gradient of 60 °C min^−1^ and a hold of 2 min. Compounds were identified by comparing their retention times and mass spectra to those of authentic standards, or by reference spectra in the Wiley and NIST libraries.

### Sequence analysis and phylogenetic tree construction

For the estimation of a phylogenetic tree, we used the MUSCLE algorithm (gap open, −2.9; gap extend, 0; hydrophobicity multiplier, 1.2; max. iterations, 8; clustering method, upgmb) implemented in MEGA7 [89] to compute an amino acid alignment. Based on the MUSCLE alignment, the tree was constructed with MEGA7 using a Maximum Likelihood algorithm (Poisson model). All positions with less than 80% site coverage were eliminated. A bootstrap resampling analysis with 1000 replicates was performed to evaluate the tree topology. For the phylogenetic tree, we included identified and characterized terpene synthases from plant-associated Ascomycota.

### Accession numbers

Sequence data for *CxTPS1* (MW331493) and *CxTPS2* (MW331494) can be found in the NCBI GenBank [85] under the corresponding identifiers. Raw reads of the RNAseq experiment were deposited in the NCBI Sequence Read Archive under the BioProject accession PRJNA682522.

## Supporting Information

File 1 Sequences of isolated endophytic fungi and identification according to NCBI database, primer used in this study, representative total ion chromatograms of single endophytic volatile blend, mass spectra of unknown volatile organic compounds, and BUSCO analysis of *Cladosporium* sp. de novo assembly.
