# Supplementary material for: Volatile emission and biosynthesis in endophytic fungi colonizing black poplar leaves
Source: Beilstein J Org Chem. 2021 Jul 22;17:1698–711. doi: 10.3762/bjoc.17.118 (PMC8313976; doi:10.3762/bjoc.17.118)
Supplement: File 1 — Sequences of isolated endophytic fungi and identification according to NCBI database, primer used in this study, representative total ion chromatograms of single endophytic volatile blend, mass spectra of unknown volatile organic compounds, and BUSCO analysis of Cladosporium sp. de novo assembly. [file Beilstein_J_Org_Chem-17-1698-s001.pdf]

BEILSTEIN JOURNAL OF ORGANIC CHEMISTRY

Supporting Information

for

Volatile emission and biosynthesis in endophytic fungi colonizing black poplar leaves

Christin Walther, Pamela Baumann, Katrin Luck, Beate Rothe, Peter H. W. Biedermann, Jonathan Gershenzon, Tobias G. Köllner and Sybille B. Unsicker

Beilstein J. Org. Chem. **2021,** *17,* 1698–1711. doi:10.3762/bjoc.17.118

Sequences of isolated endophytic fungi and identification according to NCBI database, primer used in this study, representative total ion chromatograms of single endophytic volatile blend, mass spectra of unknown volatile organic compounds, and BUSCO analysis of *Cladosporium* sp. de novo assembly

License and Terms: This is a supporting information file under the terms of the Creative Commons Attribution License (https://creativecommons.org/licenses/by/4.0). Please note that the reuse, redistribution and reproduction in particular requires that the author(s) and source are credited and that individual graphics may be subject to special legal provisions.
The license is subject to the *Beilstein Journal of Organic Chemistry* terms and conditions: (https://www.beilstein-journals.org/bjoc/terms)

Contents

Table S1: Sequences of isolated endophytic fungi and identification according to NCBI database

Table S2: Primer used in this study

Table S3: Numbers for volatile organic compounds shown on total ion chromatograms in Figure S1

Figure S1: Representative total ion chromatograms of the volatiles measured from endophytes in this study.

Figure S2: Mass spectra of unknown volatile organic compounds

Figure S3: BUSCO analysis of *Cladosporium* sp. de novo assembly

Table S1: Endophytes were identified to genus level via sequencing of ribosomal DNA (ITS1F/ ITS4). The obtained sequences were compared to the NCBI sequence database and the identity (%) of best hits with their accession number is given in the main document.

**Species**	**Sequence**
Alternaria infectoria	TGTCTTTTGCGTACTTCTTGTTTCCTGGGTGGGCTCGCCCGCCCTCAGGACCAAC<br>CACAAACCTTTTGCAATAGCAATCAGCGTCAGTAACAACGTAATTAATTACAACTT<br>TCAACAACGGATCTCTTGGTTCTGGCATCGATGAAGAACGCAGCGAAATGCGATA<br>CGTAGTGTGAATTGCAGAATTCAGTGAATCATCGAATCTTTGAACGCACATTGCG<br>CCCTTTGGTATTCCAAAGGGCATGCCTGTTCGAGCGTCATTTGTACCCTCAAGCT<br>TTGCTTGGTGTTGGGCGTCTTTTGTCTCCAGTTCGCTGGAGACTCGCCTTAAAGT<br>CATTGGCAGCCGGCCTACTGGTTTCGGAGCGCAGCACAAGTCGCGCTCTTCGCC<br>AGCCAAGGTCAGCGTCCAGCAAGCCTTTTTTTCAACCTTTGACCTCGGATCAGGT<br>AGGGATACCCG
Alternaria sp. 1	TTCTTGTTTCCTTGGTGGGTTCGCCCACCACTAGGACAAACATAAACCTTTTGTAA<br>TTGCAATCAGTGTCAGTAACAAATTAATAATTACAACTTTCAACAACGGATCTCTT<br>GGTTCTGGCATCGATGAAGAACGCAGCGAAATGCGATAAGTAGTGTGAATTGCA<br>GAATTCAGTGAATCATCGAATCTTTGAACGCACATTGCGCCCTTTGGTATTCCAAA<br>GGGCATGCCTGTTCGAGCGTCATTTGTACCCTCAAGCTTTGCTTGGTGTTGGGC<br>GTCTTGTCTCTAGCTTTGCTGGAGACTCGCCTTAAAGTAATTGGCAGCCGGCCTA<br>CTGGTTTCGGAGCGCAGCACAAGTCGCACTCTCTATCAGCAAAGGTCTAGCATC<br>CATTAAGCCTTTTTTTCAACTTTTGACCTCGGATCAGGTAGGGATACCCGCTGAAC<br>TTAAGCATATCAATAAGCGGAGGA
Stemphylium sp.	AAAAATGTGGTCTTGATGGATGCTCAACCAAGGCCGATTCAAAGTGCAAGAATTG<br>TGCTGCGCTCCGAAACCAGTAGGTCGGCTGCCAATCATTTTAAGGCGAGTCTCG<br>TGAGAGACAAAGACGCCCAACACCAAGCAAAGCTTGAGGGTACAAATGACGCTC<br>GAACAGGCATGCCCTTTGGAATACCAAAGGGCGCAATGTGCGTTCAAAGATTCG<br>ATGATTCACTGAATTCTGCAATTCACACTACGTATCGCATTTCGCTGCGTTCTTCA<br>TCGATGCCAGAACCAAGAGATCCGTTGTTGAAAGTTGTAATAATTACATTGTTTAC<br>TGACGCTGATTGCAATTACAAAAAGGTTTATGGTTTGGTCCTGGTGGCGGGCGAA<br>CCCGCCCAGGAAACAAGAAGTGCGCAAAAGACATGGGTGAATAATTCAGACAAG<br>CTGGAGCCCTCACCGAGGTGAGGTCCCAACCCGCTTTCATATTGTGTAATGATCC<br>CTCCGCAGGTTCACC
Aureobasidium sp. 1	GTCCCAGGCGAGCGCCCGCCAGAGTTAAACCAAACTCTTGTTATTTAACCGGTC<br>GTCTGAGTTAAAATTTTGAATAAATCAAAACTTTCAACAACGGATCTCTTGGTTCTC<br>GCATCGATGAAGAACGCAGCGAAATGCGATAAGTAATGTGAATTGCAGAATTCAG<br>TGAATCATCGAATCTTTGAACGCACATTGCGCCCCTTGGTATTCCGAGGGGCATG<br>CCTGTTCGAGCGTCATTACACCACTCAAGCTATGCTTGGTATTGGGCGTCGTCCT<br>TAGTTGGGCGCGCCTTAAAGACCTCGGCGAGGCCACTCCGGCTTTAGGCGTAGT<br>AGAATTTATTCGAACGTCTGTCAAAGGAGAGGAACTCTGCCGACTGAAACCTTTA<br>TTTTTCTAGGTTGACCTCGGATCAGGTAGGGATACCC

Species	Sequence
Aureobasidium sp. 2	ATAAAGGTTTCAGTCGGCAGAGTTCCTCTCCTTTGACAGACGTTCGAATAAATTCT<br>ACTACGCCTAAAGCCGGAGTGGCCTCGCCGAGGTCTTTAAGGCGCGCCCAACTA<br>AGGACGACGCCCAATACCAAGCATAGCTTGAGTGGTGTAATGACGCTCGAACAG<br>GCATGCCCCTCGGAATACCAAGGGGCGCAATGTGCGTTCAAAGATTCGATGATT<br>CACTGAATTCTGCAATTCACATTACTTATCGCATTTCGCTGCGTTCTTCATCGATG<br>CGAGAACCAAGAGATCCGTTGTTGAAAGTTTTGATTTATTCAAAATTTTAACTCAG<br>ACGACCGGTTAAATAACAAGAGTTTGGTTTAACTCTGGCGGGCGCTCGCCTGGG<br>ACGAATCCCCAGCGGCTCGAGACCGAGCGGTCCCGCCAAAGCAACAAGGTAGTT<br>TTAACAACAAAGGGTTGGAGGTCGGGCGCTGAGCACCCTTACTCTTTAATGATCC<br>TTCCGCAGGTTCACCTACGGAAGNGGATNATTAAAGAGTAAGGGTGCTCAGCGC<br>CCGACCTCCAACCCTTTGTTGTTAAAACTACCTTGTTGCTTTGGCGGGACCGCTC<br>GGTCTCGAGCCGCTGGGATTCGTCCCAGGCGAGCGCCCGCCAGAGTTAAACCA<br>AACTCTTGTTATTTAACCGGTCGTCTGAGTTAAAATTTNGAATAAATNAAAACTTTN<br>ACAACGGANCTCTTGGTTCTCGCATCGA
Didymella glomerata	CCGCCGATTGGNCAATTTAAACNATTTGCAGTTGCAATCAGCGTCTGAAAAAACT<br>TAATAGTTACAACTTTCAACAACGGATCTCTTGGTTCTGGCATCGATGAAGAACGC<br>AGCGAAATGCGATAAGTAGTGTGAATTGCAGAATTCAGTGAATCATCGAATCTTT<br>GAACGCACATTGCGCCCCTTGGTATTCCATGGGGCATGCCTGTTCGAGCGTCAT<br>TTGTACCTTCAAGCTCTGCTTGGTGTTGGGTGTTTGTCTCGCCTCTGCGTGTAGA<br>CTCGCCTCAAAACAATTGGCAGCCGGCGTATTGATTTCGGAGCGCAGTACATCTC<br>GCGCTTTGCACTCATAACGACGACGTCCAAAAGTACATTTTTACACTCTTGACCTC<br>GGATCAGGTAGGGATACCCGCTGAACTTAAGCATATCAATAAGCGG
Didymella sp. 1	CCGCCGATTGGACAATTTAAACCATTTGCAGTTGCAATCAGCGTCTGAAAAAACTT<br>AATAGTTACAACTTTCAACAACGGATCTCTTGGTTCTGGCATCGATGAAGAACGC<br>AGCGAAATGCGATAAGTAGTGTGAATTGCAGAATTCAGTGAATCATCGAATCTTT<br>GAACGCACATTGCGCCCCTTGGTATTCCATGGGGCATGCCTGTTCGAGCGTCAT<br>TTGTACCTTCAAGCTCTGCTTGGTGTTGGGTGTTTGTCTCGCCTCTGCGTGTAGA<br>CTCGCCTCAAAACAATTGGCAGCCGGCGTATTGATTTCGGAGCGCAGTACATCTC<br>G
Didymella sp. 2	CTTTTAAGTACCTTACGTTTCCTCGGCGGGTCCGCCCGCCGATTGGACAATTTAA<br>ACCATTTGCAGTTGCAATCAGCGTCTGAAAAAACTTAATAGTTACAACTTTCAACA<br>ACGGATCTCTTGGTTCTGGCATCGATGAAGAACGCAGCGAAATGCGATAAGTAGT<br>GTGAATTGCAGAATTCAGTGAATCATCGAATCTTTGAACGCACATTGCGCCCCTT<br>GGTATTCCATGGGGCATGCCTGTTCGAGCGTCATTTGTACCTTCAAGCTCTGCTT<br>GGTGTTGGGTGTTTGTCTCGCCTCTGCGTGTAGACTCGCCTCAAAACAATTGGCA<br>GCCGGCGTATTGATTTCGGAGCGCAGTACATCTCGCGCTTTGCACTCATAACGAC<br>GACGTCCAAAAGTACATT
Cladosporium sp.	TCGGGCGGGGGCTCCGGGTGGACACTTCAAACTCTTGCGTAACTTTGCAGTCTG<br>AGTAAACTTAATTAATAAATTAAAACTTTTAACAACGGATCTCTTGGTTCTGGCATC<br>GATGAAGAACGCAGCGAAATGCGATAAGTAATGTGAATTGCAGAATTCAGTGAAT<br>CATCGAATCTTTGAACGCACATTGCGCCCCCTGGTATTCCGGGGGGCATGCCTG<br>TTCGAGCGTCATTTCACCACTCAAGCCTCGCTTGGTATTGGGCAACGCG
Fusarium sp.	GGGACGGCCCGCCGCAGGAAACCCTAAACTCTGTTTTTAGTGGAACTTCTGAGT<br>ATAAAAAACAAATAAATCAAAACTTTCAACAACGGATCTCTTGGTTCTGGCATCGA<br>TGAAGAACGCAGCAAAATGCGATAAGTAATGTGAATTGCAGAATTCAGTGAATCA<br>TCGAATCTTTGAACGCACATTGCGCCCGCCAGTATTCTGGCGGGCATGCCTGTTC<br>GAGCGTCATTTCAACCCTCAAGCCCAGCTTGGTGTTGGGATCTGTGTGCAAACAC<br>AGTCCCCAAATTGATTGGCGGTCACGTCGAGCTTCCATAGCGTAGTA
Sordaria sp.	CGGGCCCCCGGATCCTCGGGTCTCCCGCTCGCGGGAGGCTGCCCGCCGGAGT<br>GCCGAAACCAAACTCTTGATATTTTATGTCTCTCTGAGTAAACTTTTAAATAAGTCA<br>AAACTTTCAACAACGGATCTCTTGGTTCTGGCATCGATGAAGAACGCAGCGAAAT<br>GCGATAAGTAATGTGAATTGCAGAATTCAGTGAATCATCGAATCTTTGAACGCACA<br>TTGCGCTCGCCAGTATTCTGGCGAGCATGCCTGTTCGAGCGTCATTTCAACCATC<br>AAGCTCTGCTTGCGTTGGGGATCCGCGTCTGACGCGGTCCCTCAAAAACAGTGG<br>CGGGCTCGCTAGTCACACCGAGCGTAGTAACTCTACATCGCTATGGTCGTGCGG<br>CGGGTTCTTGCCGTAAAACCCCCAATTTCTAAGGTTGACCTCGGATCAGGTAGGA<br>ATACCCGCTGAACTTAAGCATATCAATAAGCGGAGGA

Species	Sequence
Arthrinium sp.	AAAAATGTGGTCTTGATGGATGCTCAACCAAGGCCGATTCAAAGTGCAAGAATTG<br>TGCTGCGCTCCGAAACCAGTAGGTCGGCTGCCAATCATTTTAAGGCGAGTCTCG<br>TGAGAGACAAAGACGCCCAACACCAAGCAAAGCTTGAGGGTACAAATGACGCTC<br>GAACAGGCATGCCCTTTGGAATACCAAAGGGCGCAATGTGCGTTCAAAGATTCG<br>ATGATTCACTGAATTCTGCAATTCACACTACGTATCGCATTTCGCTGCGTTCTTCA<br>TCGATGCCAGAACCAAGAGATCCGTTGTTGAAAGTTGTAATAATTACATTGTTTAC<br>TGACGCTGATTGCAATTACAAAAAGGTTTATGGTTTGGTCCTGGTGGCGGGCGAA<br>CCCGCCCAGGAAACAAGAAGTGCGCAAAAGACATGGGTGAATAATTCAGACAAG<br>CTGGAGCCCTCACCGAGGTGAGGTCCCAACCCGCTTTCATATTGTGTAATGATCC<br>CTCCGCAGGTTCACC

Table S2: Primers used in this study.

Name	Sequence	Purpose
CxTPS1_Fwd	CACCATGAGCTCTAGCACGGGTC	cloning
CxTPS1_Rev	TCACGACGCCCCTCG	cloning
CxTPS2_Fwd	CACCATGTCAGACCCTACTCGCC	cloning
CxTPS2_Rev	TCAGCAACACTCCAGATAGCTAGG	cloning
ITS1F	CTTGGTCATTTAGAGGAAGTAA	amplify fungal rRNA ITS
ITS4	TCCTCCGCTTATTGATATGC	amplify fungal rRNA ITS

Table S3: Numbers for volatile organic compounds, which are shown in the total ion chromatograms of the volatile bouquet for each endophytic fungus in Figure S1.

#	Volatile organic compound	R.T. (min)
1	Ethanol	1.525
2	2-Butanone	1.855
3	Ethyl Acetate	1.940
4	2-Methyl-1-propanol	2.020
5	unknown 1	2.265
6	3-Hydroxy-2-Butanone	2.720
7	3-Methyl-1-butanol	3.035
8	2-Methyl-1-butanol	3.070
9	unknown 2	3.780
10	3-Methylbutyl acetate	6.100
11	Ethenylbenzene	6.325
12	unknown 3	6.730
13	unknown 4	10.380
14	unknown 5	10.655
15	2-Phenylethanol	12.240
16	unknown 6	17.465
17	unknown 7	17.650
18	α-Cubebene	17.920

19	unknown 8	17.950
20	unknown 9	18.075
21	unknown 10	18.245
22	unknown 11	18.330
23	α-Copaene	18.525
24	unknown 12	18.765
25	unknown 13	18.850
26	Sativene	18.985
27	α-Gurjunene	19.280
28	unknown 14	19.300
29	unknown 15	19.380
30	unknown 16	19.400
31	unknown 17	19.460
32	Aristolene	19.485
33	(*E*)-β-Caryophyllene	19.500
34	unknown 18	19.535
35	unknown 19	19.675
36	unknown 20	19.695
37	Bicyclosesquiphellandrene	19.745
38	β-Gurjunene	19.775
39	unknown 21	19.790
40	unknown 22	19.845
41	unknown 23	19.880
42	α-Guaiene	20.000
43	unknown 24	20.015
44	unknown 25	20.120
45	unknown 26	20.140
46	(*E*)-β-Farnesene	20.195
47	unknown 27	20.325
48	unknown 28	20.440
49	unknown 29	20.490
50	unknown 30	20.555
51	β-Chamigrene	20.585
52	unknown 31	20.610
53	unknown 32	20.610
54	α-Selinene	20.665
55	γ-Muurolene	20.685
56	unknown 33	20.800
57	unknown 34	20.860
58	β-Selinene	20.910
59	unknown 35	20.930
60	(+)-Valencene	21.040
61	unknown 36	21.115
62	α-Muurolene	21.170
63	β-Himachalene	21.195
64	β-Bisabolene	21.305
65	unknown 37	21.630

66	unknown 38	21.635
67	unknown 39	21.775
68	unknown 40	21.985
69	unknown 41	22.075
70	unknown 42	22.150
71	unknown 43	22.355
72	unknown 44	22.720
73	unknown 45	23.235
74	unknown 46	23.875
75	unknown 47	24.485
76	unknown 48	24.675
77	unknown 49	25.980

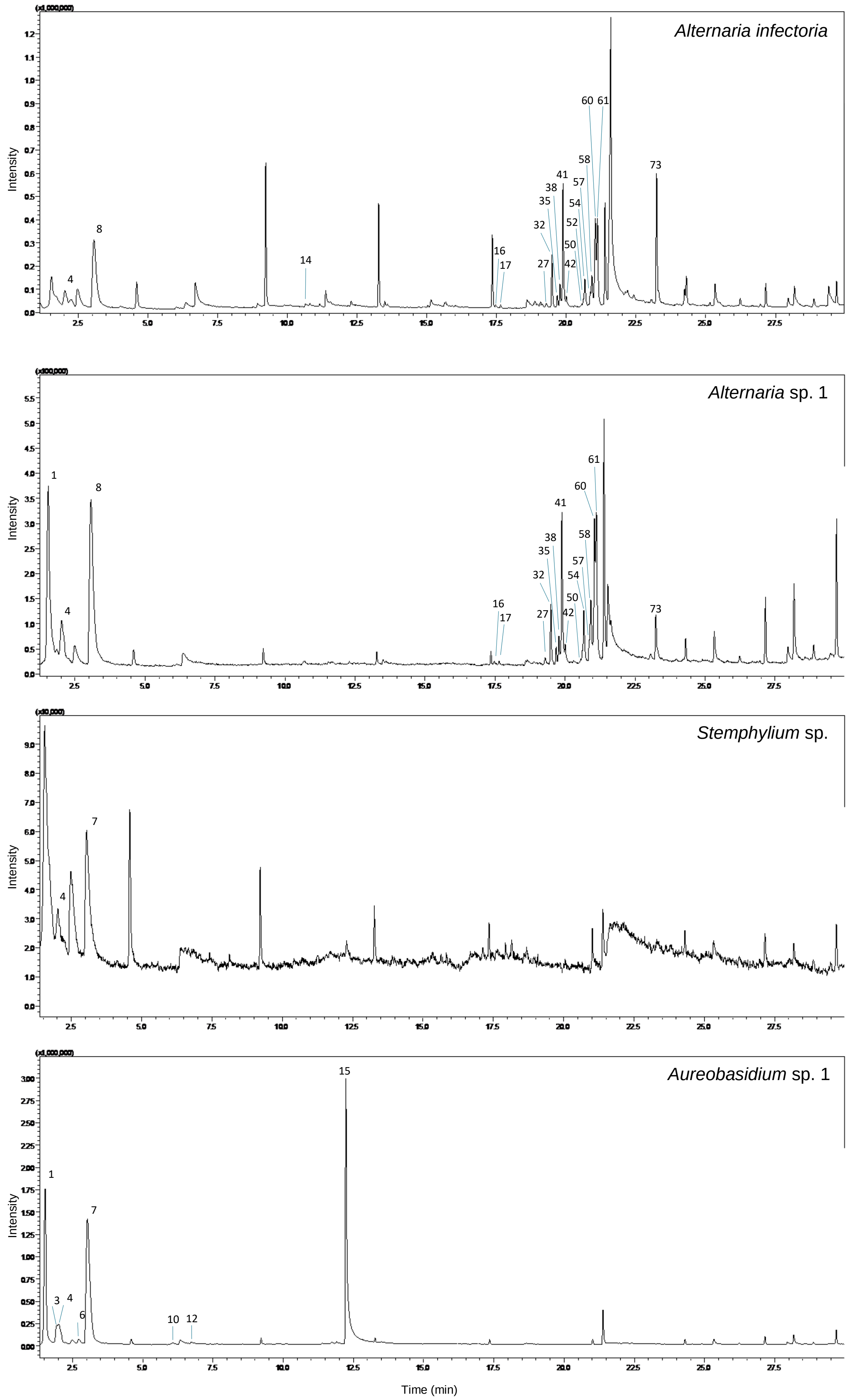

(x1,000,000)
Alternaria infectoria
Intensity
4
8
14
16
17
27
32
35
38
41
42
50
52
54
57
58
60
61
73
(x100,000)
Alternaria sp. 1
1
4
8
16
17
27
32
35
38
41
42
50
54
57
58
60
61
73
(x10,000)
Stemphylium sp.
4
7
(x1,000,000)
Aureobasidium sp. 1
1
3
4
6
7
10
12
15
Time (min)

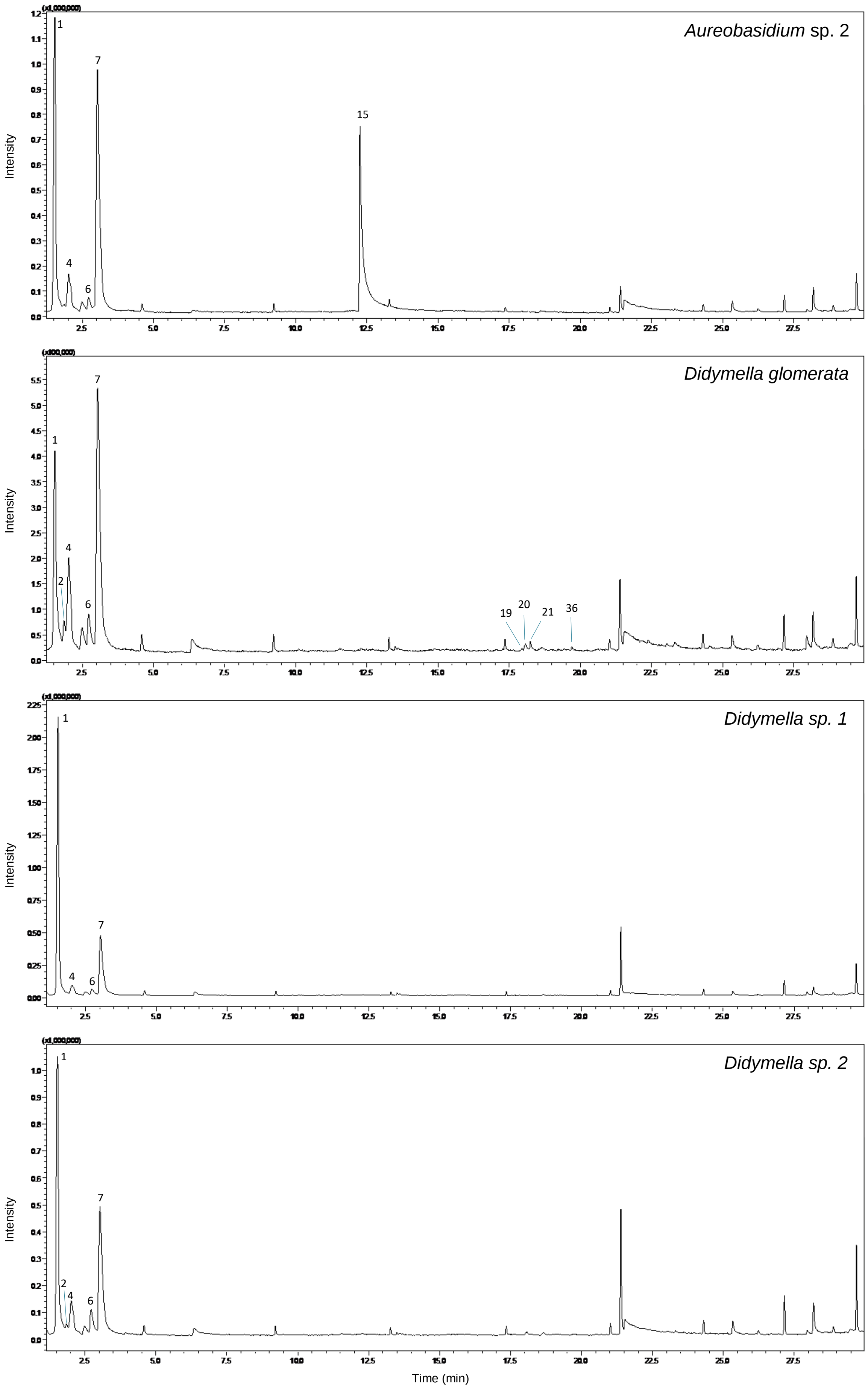

Aureobasidium sp. 2
1
7
15
4
6
Intensity
(x1,000,000)
Didymella glomerata
(x100,000)
7
1
4
2
6
19
20
21
36
Intensity
Didymella sp. 1
(x1,000,000)
1
7
4
6
Intensity
Didymella sp. 2
(x1,000,000)
1
7
2
4
6
Intensity
Time (min)

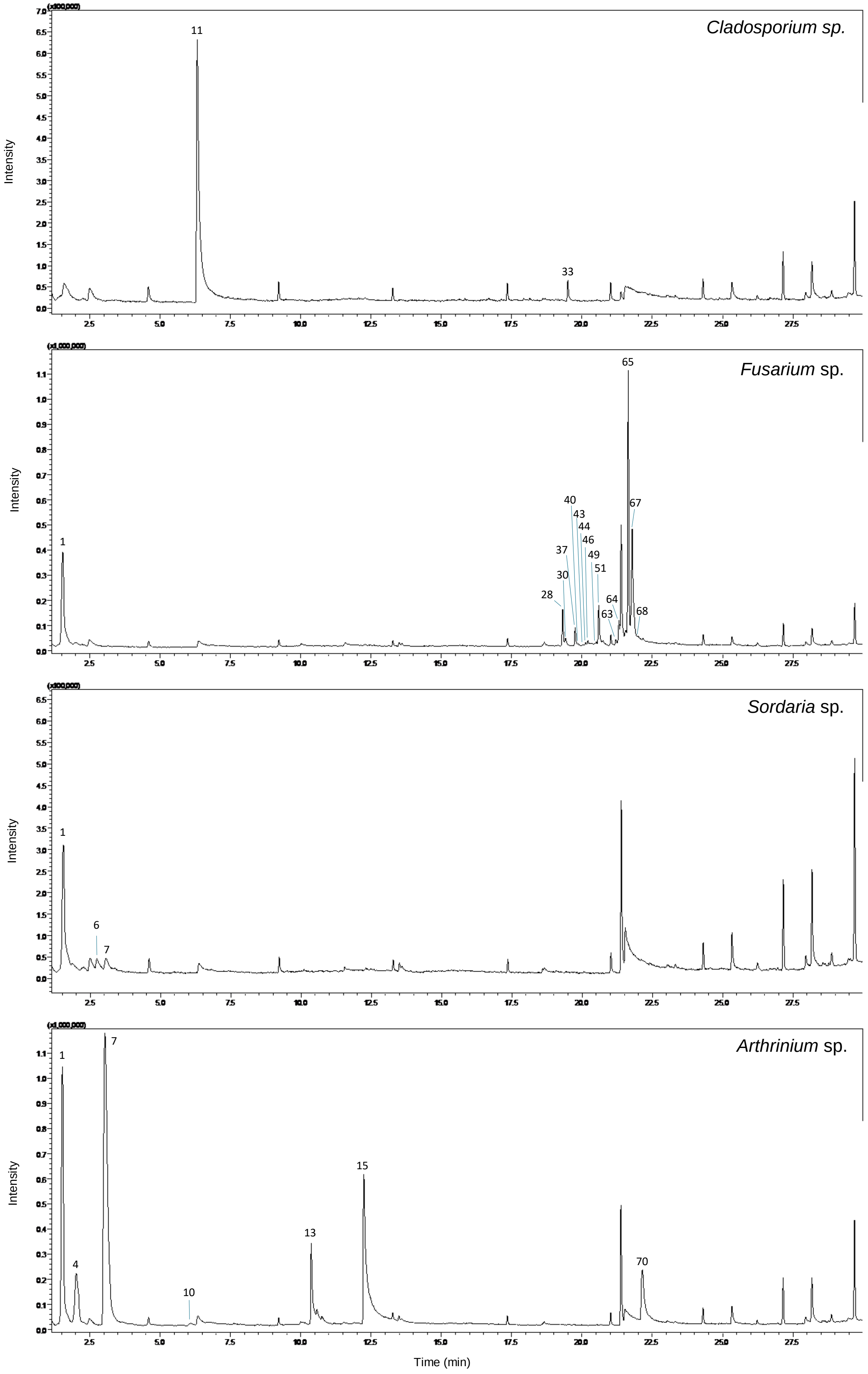
Cladosporium sp.
Fusarium sp.
Sordaria sp.
Arthrinium sp.
Intensity
Time (min)
(x100,000)
(x1,000,000)
11
33
1
28
30
37
40
43
44
46
49
51
63
64
65
67
68
6
7
4
10
13
15
70

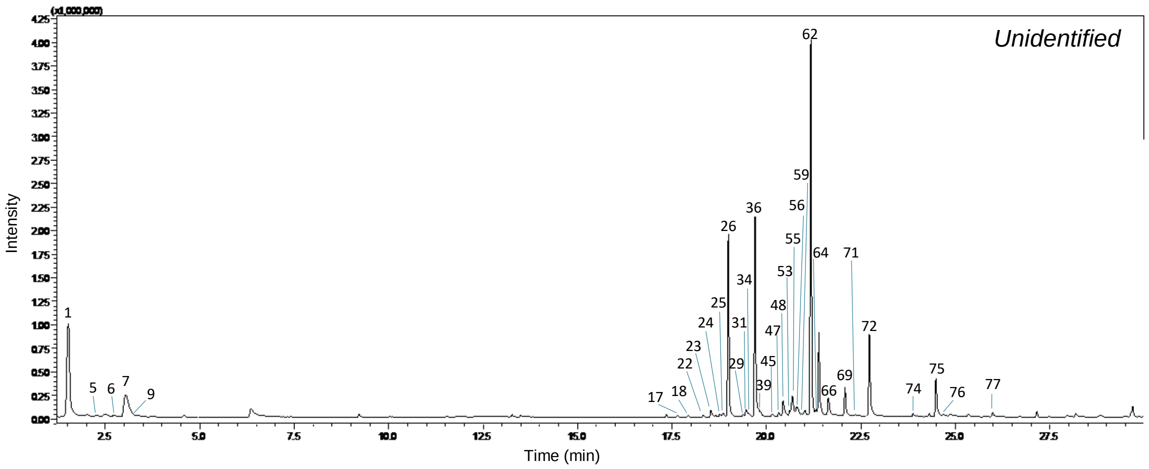

Figure S1: Representative total ion chromatogram of volatiles measured from different endophytes used in this study. Numbers indicate different volatile organic compounds, listed in Table S3. Peaks without numbers are either contamination from the PDMS tube or originate from the culture media itself.

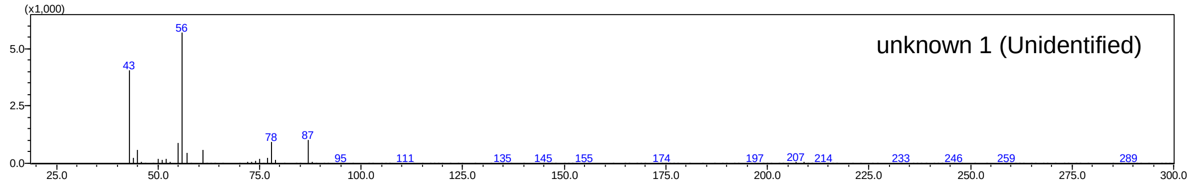

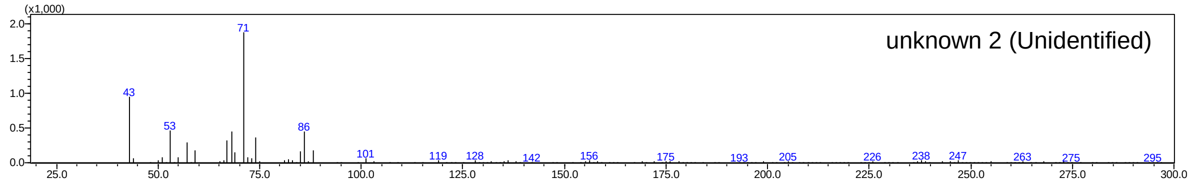

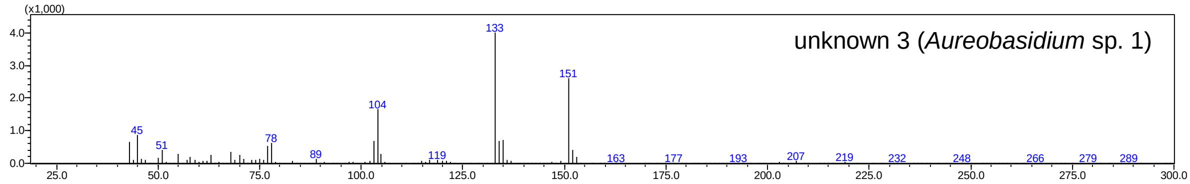

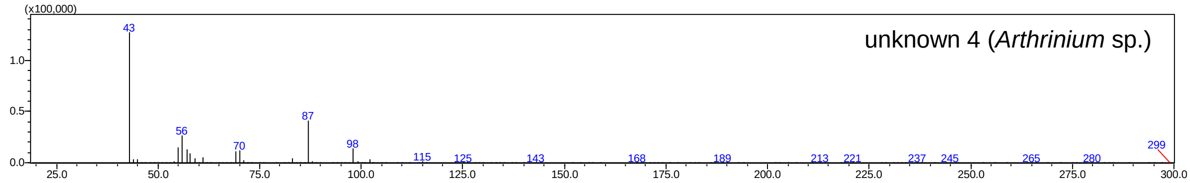

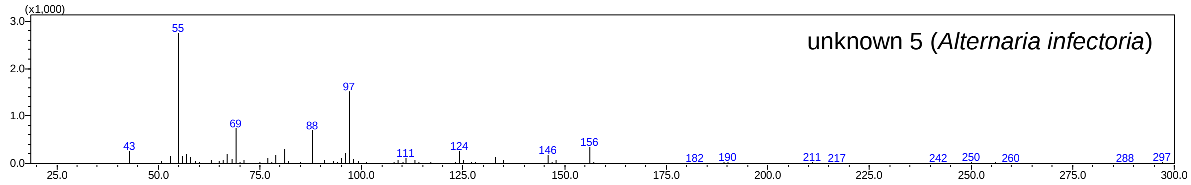
unknown 5 (Alternaria infectoria)
(x1,000)
43 55 69 88 97 111 124 146 156 182 190 211 217 242 250 260 288 297
25.0 50.0 75.0 100.0 125.0 150.0 175.0 200.0 225.0 250.0 275.0 300.0

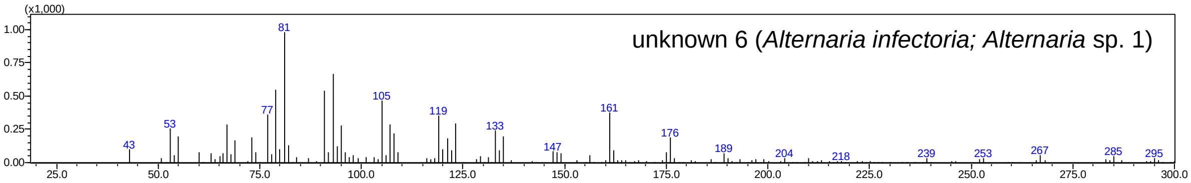
unknown 6 (Alternaria infectoria; Alternaria sp. 1)
(x1,000)
43 53 77 81 105 119 133 147 161 176 189 204 218 239 253 267 285 295
25.0 50.0 75.0 100.0 125.0 150.0 175.0 200.0 225.0 250.0 275.0 300.0

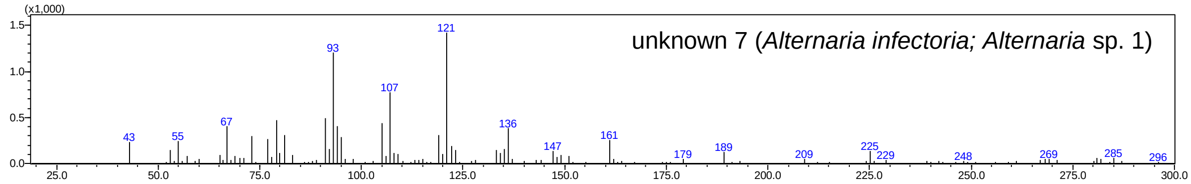
unknown 7 (Alternaria infectoria; Alternaria sp. 1)
(x1,000)
43 55 67 93 107 121 136 147 161 179 189 209 225 229 248 269 285 296
25.0 50.0 75.0 100.0 125.0 150.0 175.0 200.0 225.0 250.0 275.0 300.0

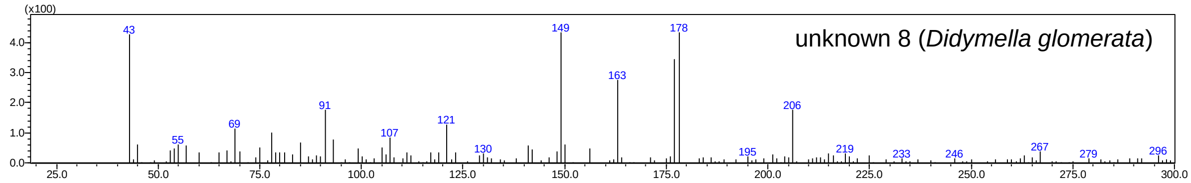
unknown 8 (Didymella glomerata)
(x100)
43 55 69 91 107 121 130 149 163 178 195 206 219 233 246 267 279 296
25.0 50.0 75.0 100.0 125.0 150.0 175.0 200.0 225.0 250.0 275.0 300.0

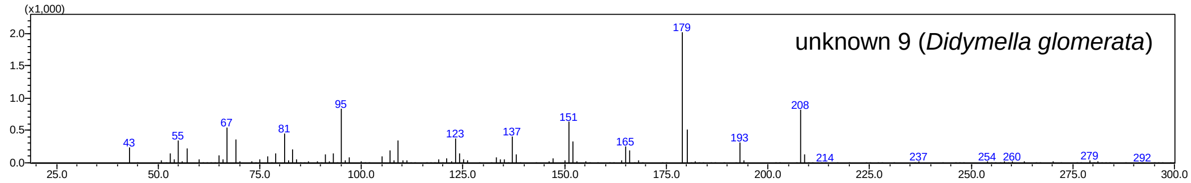
unknown 9 (Didymella glomerata)
(x1,000)
43 55 67 81 95 123 137 151 165 179 193 208 214 237 254 260 279 292
25.0 50.0 75.0 100.0 125.0 150.0 175.0 200.0 225.0 250.0 275.0 300.0

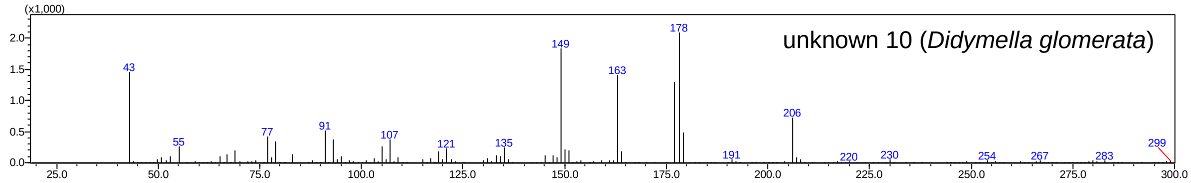
unknown 10 (Didymella glomerata)
(x1,000)
43 55 77 91 107 121 135 149 163 178 191 206 220 230 254 267 283 299
25.0 50.0 75.0 100.0 125.0 150.0 175.0 200.0 225.0 250.0 275.0 300.0

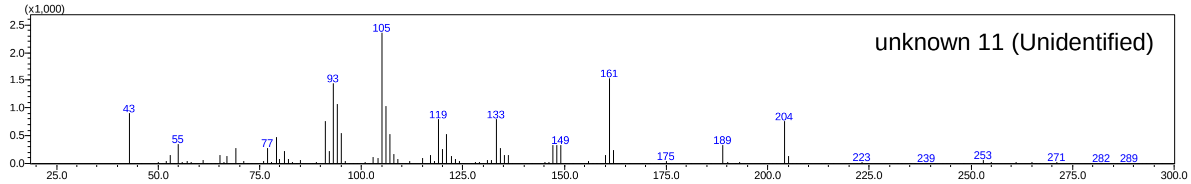
unknown 11 (Unidentified)
(x1,000)
43 55 77 93 105 119 133 149 161 175 189 204 223 239 253 271 282 289
25.0 50.0 75.0 100.0 125.0 150.0 175.0 200.0 225.0 250.0 275.0 300.0

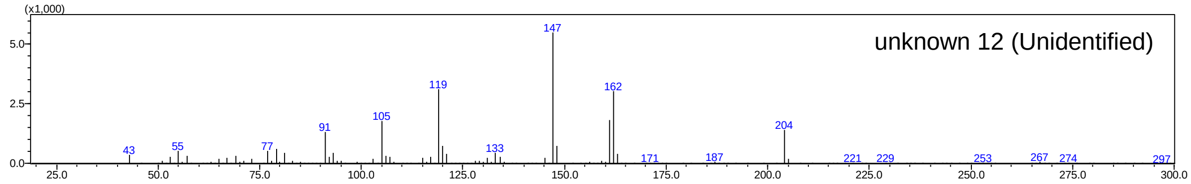
unknown 12 (Unidentified)
(x1,000)
43 55 77 91 105 119 133 147 162 171 187 204 221 229 253 267 274 297
25.0 50.0 75.0 100.0 125.0 150.0 175.0 200.0 225.0 250.0 275.0 300.0

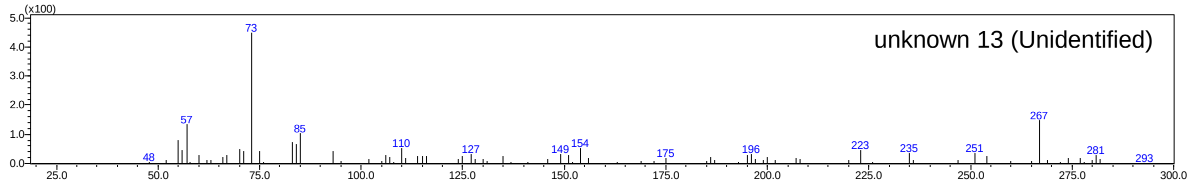
unknown 13 (Unidentified)
(x100)
73
57
85
48
110
127
149
154
175
196
223
235
251
267
281
293

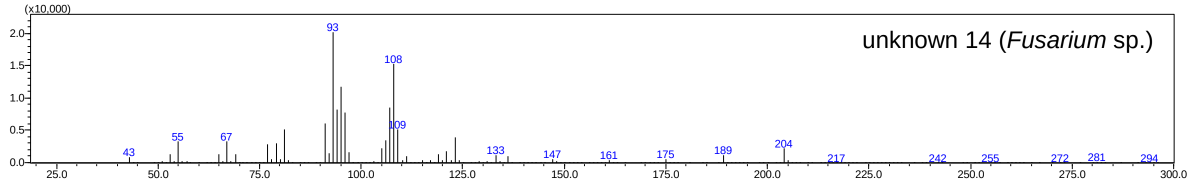
unknown 14 (*Fusarium* sp.)
(x10,000)
93
108
109
43
55
67
133
147
161
175
189
204
217
242
255
272
281
294

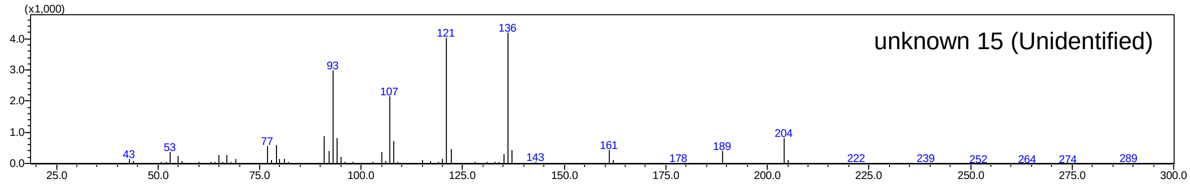
unknown 15 (Unidentified)
(x1,000)
121
136
93
107
43
53
77
143
161
178
189
204
222
239
252
264
274
289

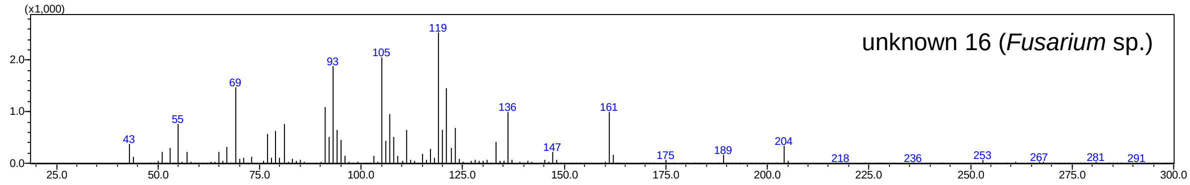
unknown 16 (*Fusarium* sp.)
(x1,000)
119
105
93
69
55
43
136
161
147
175
189
204
218
236
253
267
281
291

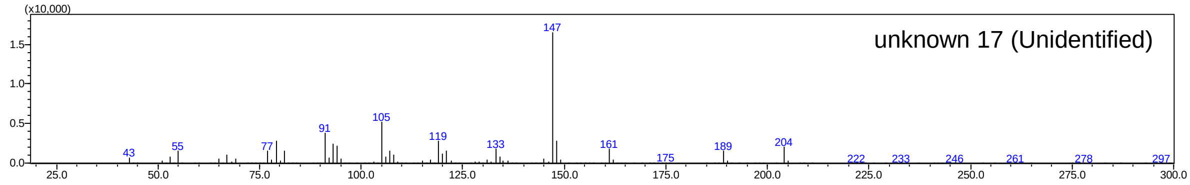
unknown 17 (Unidentified)
(x10,000)
147
105
91
119
133
43
55
77
161
175
189
204
222
233
246
261
278
297

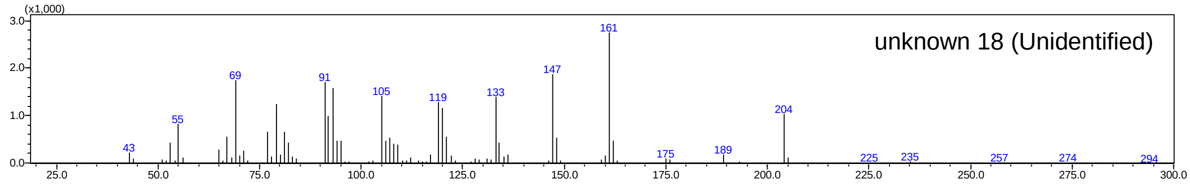
unknown 18 (Unidentified)
(x1,000)
161
147
69
91
105
119
133
55
43
204
175
189
225
235
257
274
294

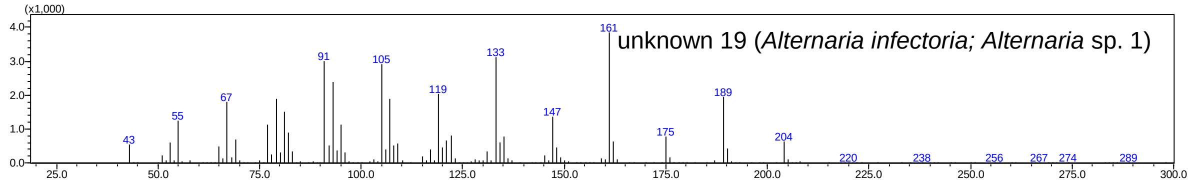
unknown 19 (*Alternaria infectoria; Alternaria* sp. 1)
(x1,000)
161
91
105
133
67
119
55
43
147
189
175
204
220
238
256
267
274
289

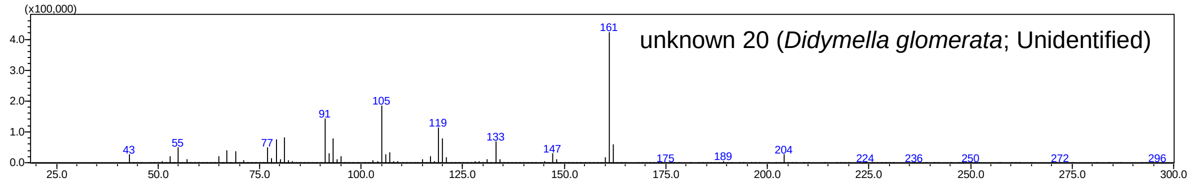
unknown 20 (*Didymella glomerata*; Unidentified)
(x100,000)
161
105
91
119
133
43
55
77
147
175
189
204
224
236
250
272
296

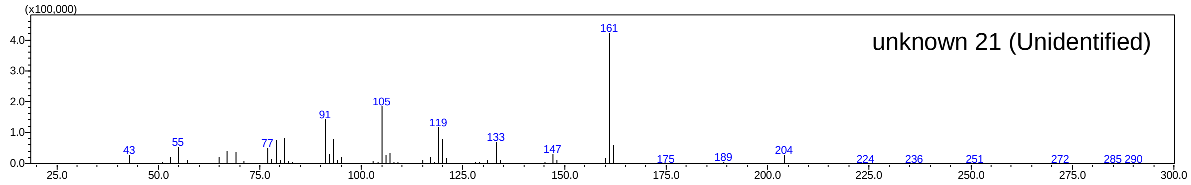
unknown 21 (Unidentified)
(x100,000)
161
105
91
119
133
43
55
77
147
175
189
204
224
236
251
272
285 290

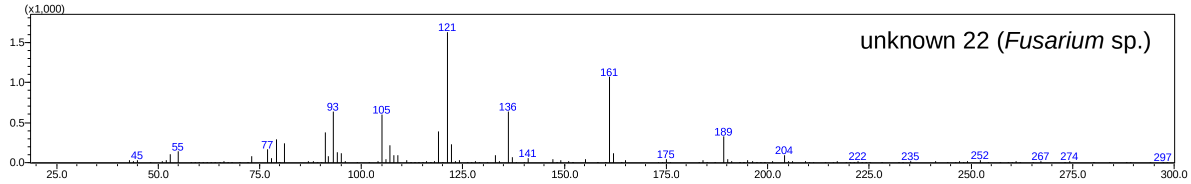
unknown 22 (*Fusarium* sp.)
(x1,000)
121
161
93
105
136
189
45
55
77
141
175
204
222
235
252
267
274
297

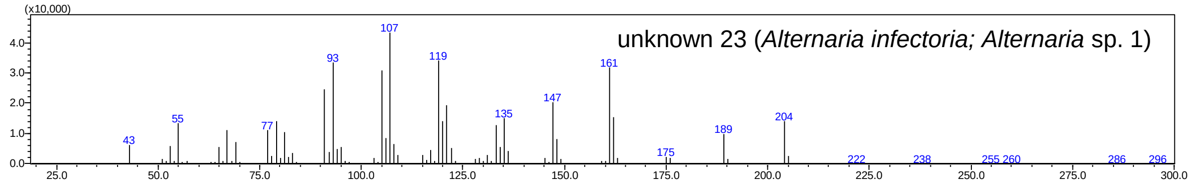
unknown 23 (*Alternaria infectoria; Alternaria* sp. 1)
(x10,000)
107
93
119
161
147
135
55
77
43
189
204
175
222
238
255 260
286
296

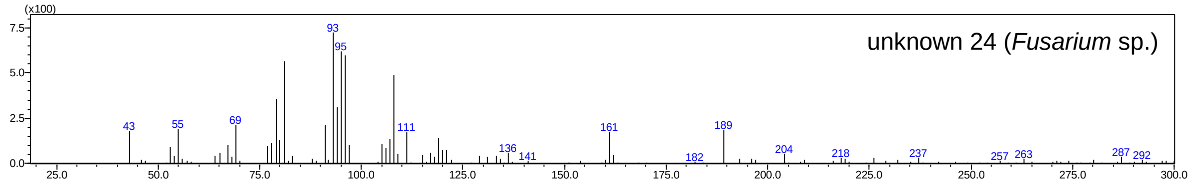
unknown 24 (*Fusarium* sp.)
(x100)
93
95
43
55
69
111
136
141
161
182
189
204
218
237
257
263
287
292

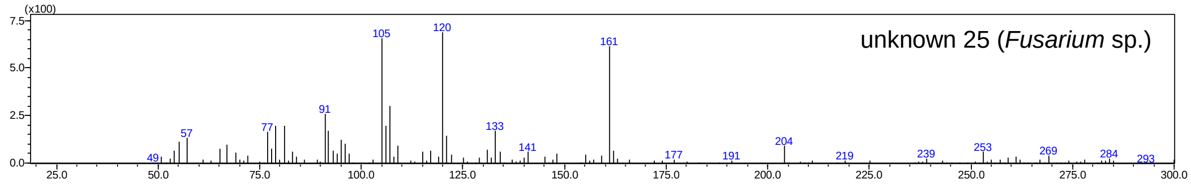
unknown 25 (*Fusarium* sp.)
(x100)
120
105
161
91
57
77
49
133
141
177
191
204
219
239
253
269
284
293

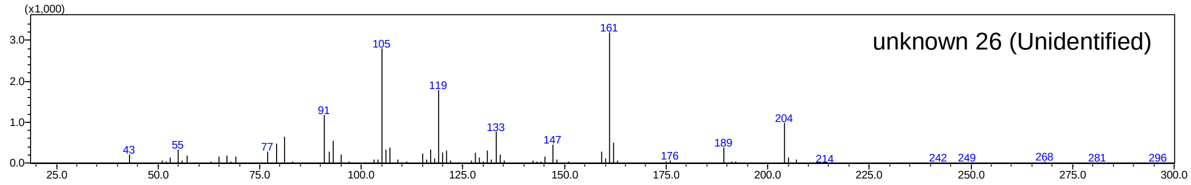
unknown 26 (Unidentified)
(x1,000)
161
105
119
91
133
147
43
55
77
176
189
204
214
242
249
268
281
296

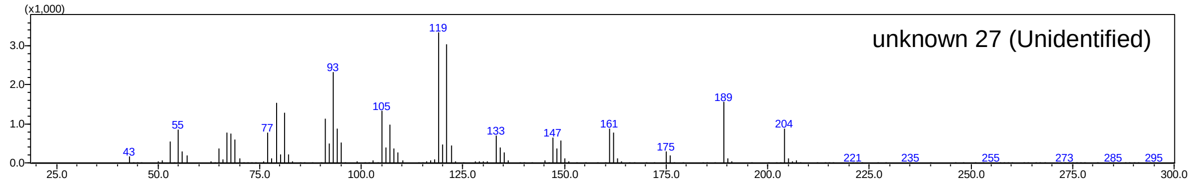
unknown 27 (Unidentified)
(x1,000)
119
93
105
189
55
77
43
133
147
161
175
204
221
235
255
273
285
295

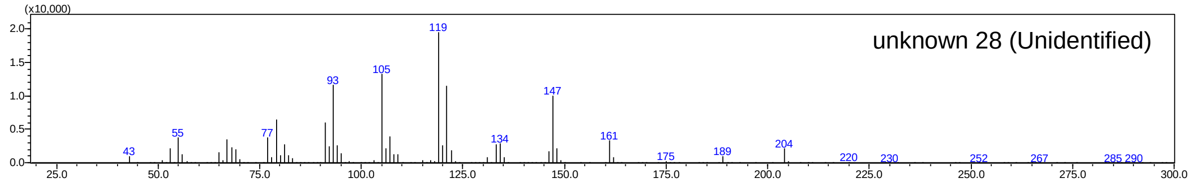
unknown 28 (Unidentified)
(x10,000)
119
93
105
147
43
55
77
134
161
175
189
204
220
230
252
267
285 290

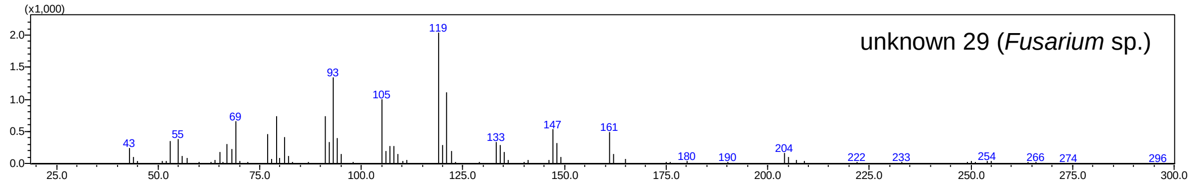
unknown 29 (*Fusarium* sp.)
(x1,000)
0.0 0.5 1.0 1.5 2.0
43 55 69 93 105 119 133 147 161 180 190 204 222 233 254 266 274 296
25.0 50.0 75.0 100.0 125.0 150.0 175.0 200.0 225.0 250.0 275.0 300.0

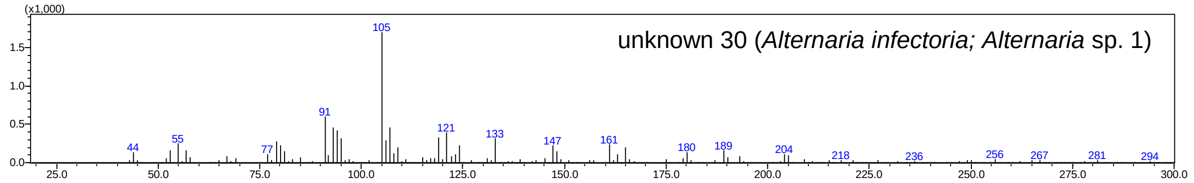
unknown 30 (*Alternaria infectoria; Alternaria* sp. 1)
(x1,000)
0.0 0.5 1.0 1.5
44 55 77 91 105 121 133 147 161 180 189 204 218 236 256 267 281 294
25.0 50.0 75.0 100.0 125.0 150.0 175.0 200.0 225.0 250.0 275.0 300.0

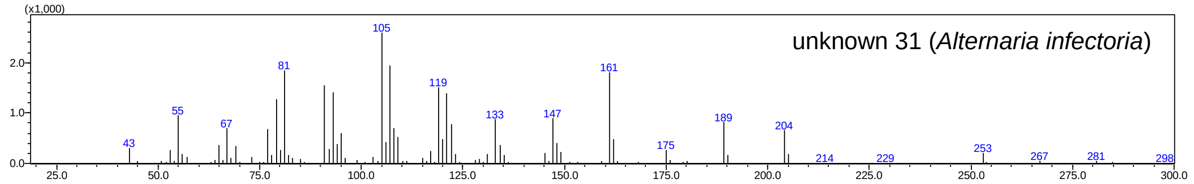
unknown 31 (*Alternaria infectoria*)
(x1,000)
0.0 1.0 2.0
43 55 67 81 105 119 133 147 161 175 189 204 214 229 253 267 281 298
25.0 50.0 75.0 100.0 125.0 150.0 175.0 200.0 225.0 250.0 275.0 300.0

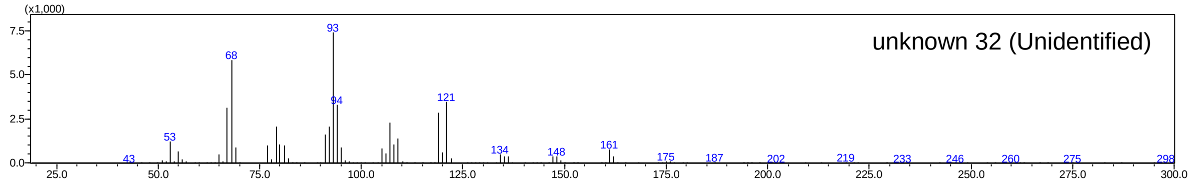
unknown 32 (Unidentified)
(x1,000)
0.0 2.5 5.0 7.5
43 53 68 93 94 121 134 148 161 175 187 202 219 233 246 260 275 298
25.0 50.0 75.0 100.0 125.0 150.0 175.0 200.0 225.0 250.0 275.0 300.0

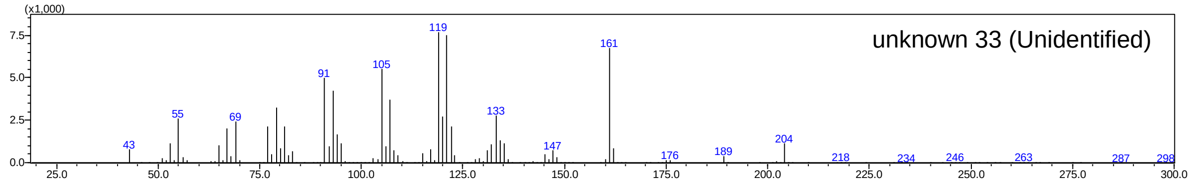
unknown 33 (Unidentified)
(x1,000)
0.0 2.5 5.0 7.5
43 55 69 91 105 119 133 147 161 176 189 204 218 234 246 263 287 298
25.0 50.0 75.0 100.0 125.0 150.0 175.0 200.0 225.0 250.0 275.0 300.0

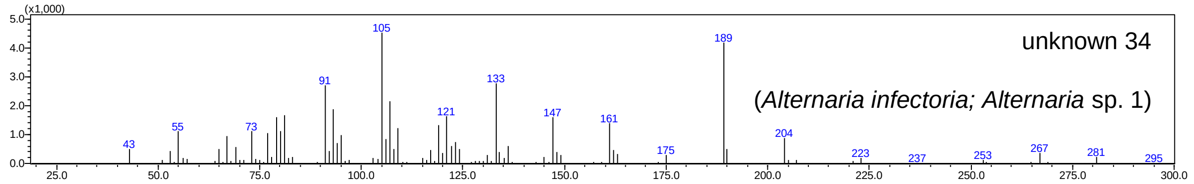
unknown 34
(*Alternaria infectoria; Alternaria* sp. 1)
(x1,000)
0.0 1.0 2.0 3.0 4.0 5.0
43 55 73 91 105 121 133 147 161 175 189 204 223 237 253 267 281 295
25.0 50.0 75.0 100.0 125.0 150.0 175.0 200.0 225.0 250.0 275.0 300.0

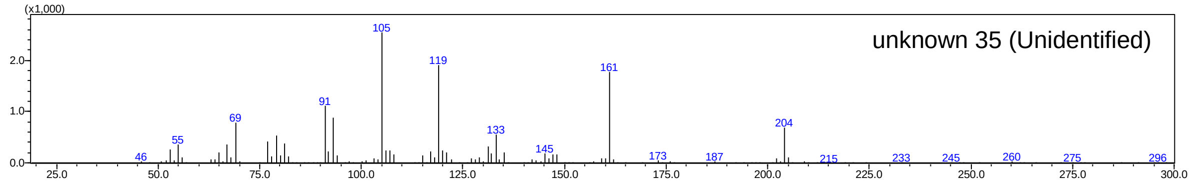
unknown 35 (Unidentified)
(x1,000)
0.0 1.0 2.0
46 55 69 91 105 119 133 145 161 173 187 204 215 233 245 260 275 296
25.0 50.0 75.0 100.0 125.0 150.0 175.0 200.0 225.0 250.0 275.0 300.0

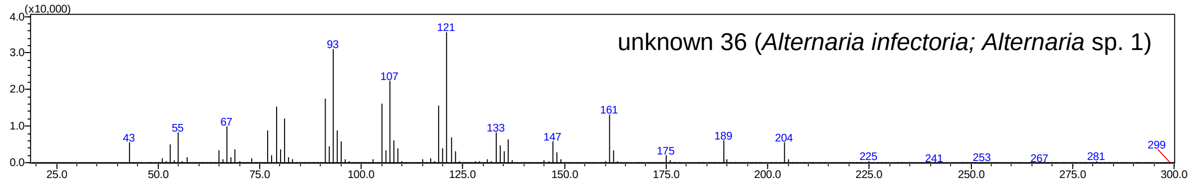
unknown 36 (*Alternaria infectoria; Alternaria* sp. 1)
(x10,000)
0.0 1.0 2.0 3.0 4.0
43 55 67 93 107 121 133 147 161 175 189 204 225 241 253 267 281 299
25.0 50.0 75.0 100.0 125.0 150.0 175.0 200.0 225.0 250.0 275.0 300.0

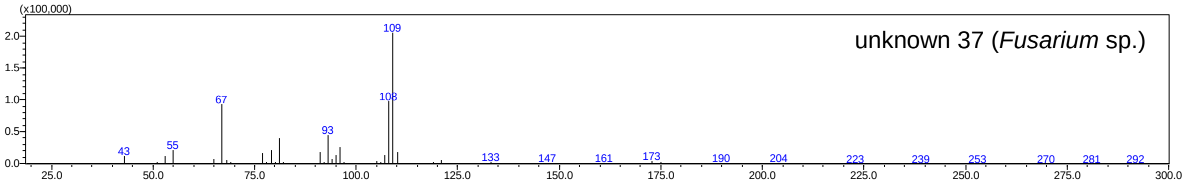
(x100,000)
unknown 37 (*Fusarium* sp.)
43 55 67 93 108 109 133 147 161 173 190 204 223 239 253 270 281 292

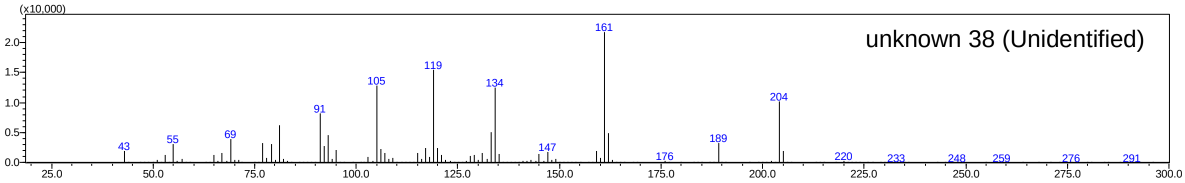
(x10,000)
unknown 38 (Unidentified)
43 55 69 91 105 119 134 147 161 176 189 204 220 233 248 259 276 291

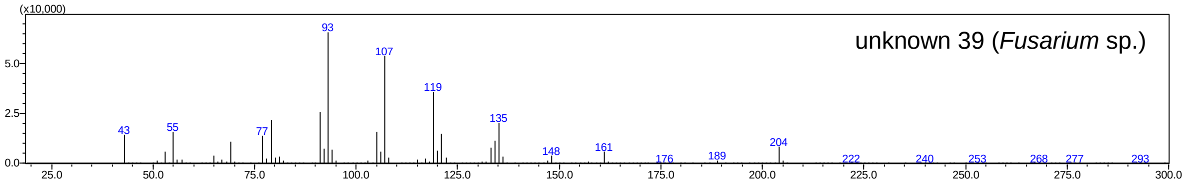
(x10,000)
unknown 39 (*Fusarium* sp.)
43 55 77 93 107 119 135 148 161 176 189 204 222 240 253 268 277 293

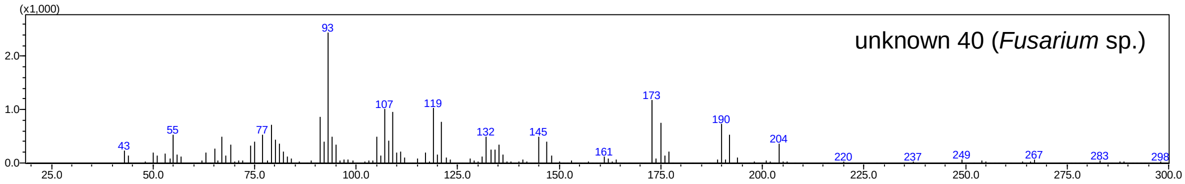
(x1,000)
unknown 40 (*Fusarium* sp.)
43 55 77 93 107 119 132 145 161 173 190 204 220 237 249 267 283 298

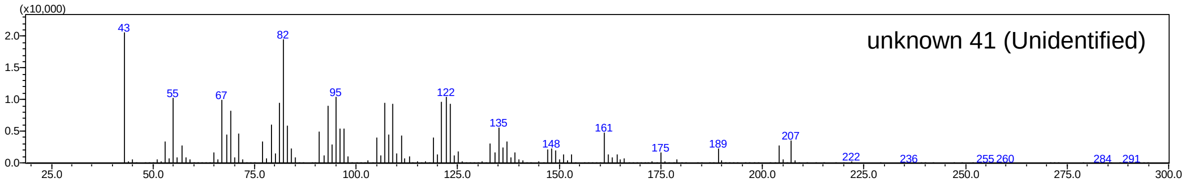
(x10,000)
unknown 41 (Unidentified)
43 55 67 82 95 122 135 148 161 175 189 207 222 236 255 260 284 291

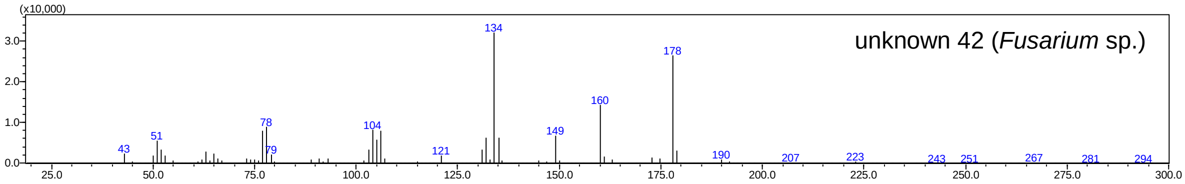
(x10,000)
unknown 42 (*Fusarium* sp.)
43 51 78 79 104 121 134 149 160 178 190 207 223 243 251 267 281 294

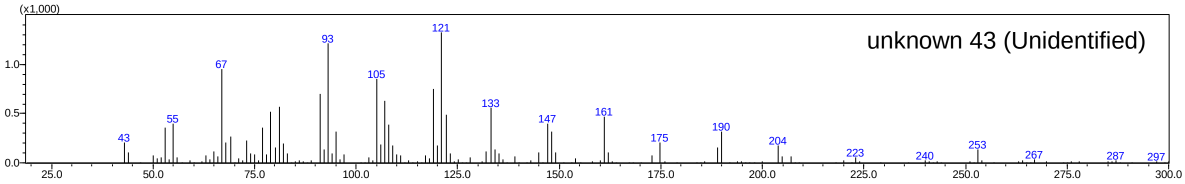
(x1,000)
unknown 43 (Unidentified)
43 55 67 93 105 121 133 147 161 175 190 204 223 240 253 267 287 297

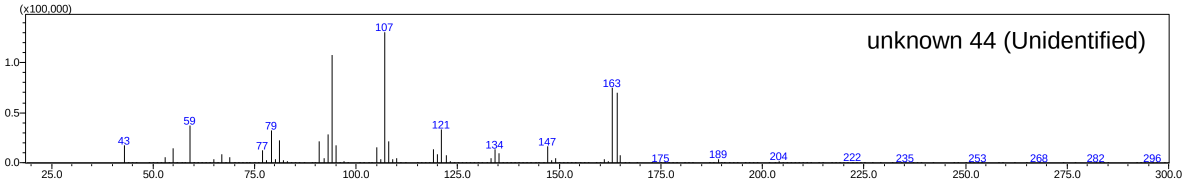
(x100,000)
unknown 44 (Unidentified)
43 59 77 79 107 121 134 147 163 175 189 204 222 235 253 268 282 296

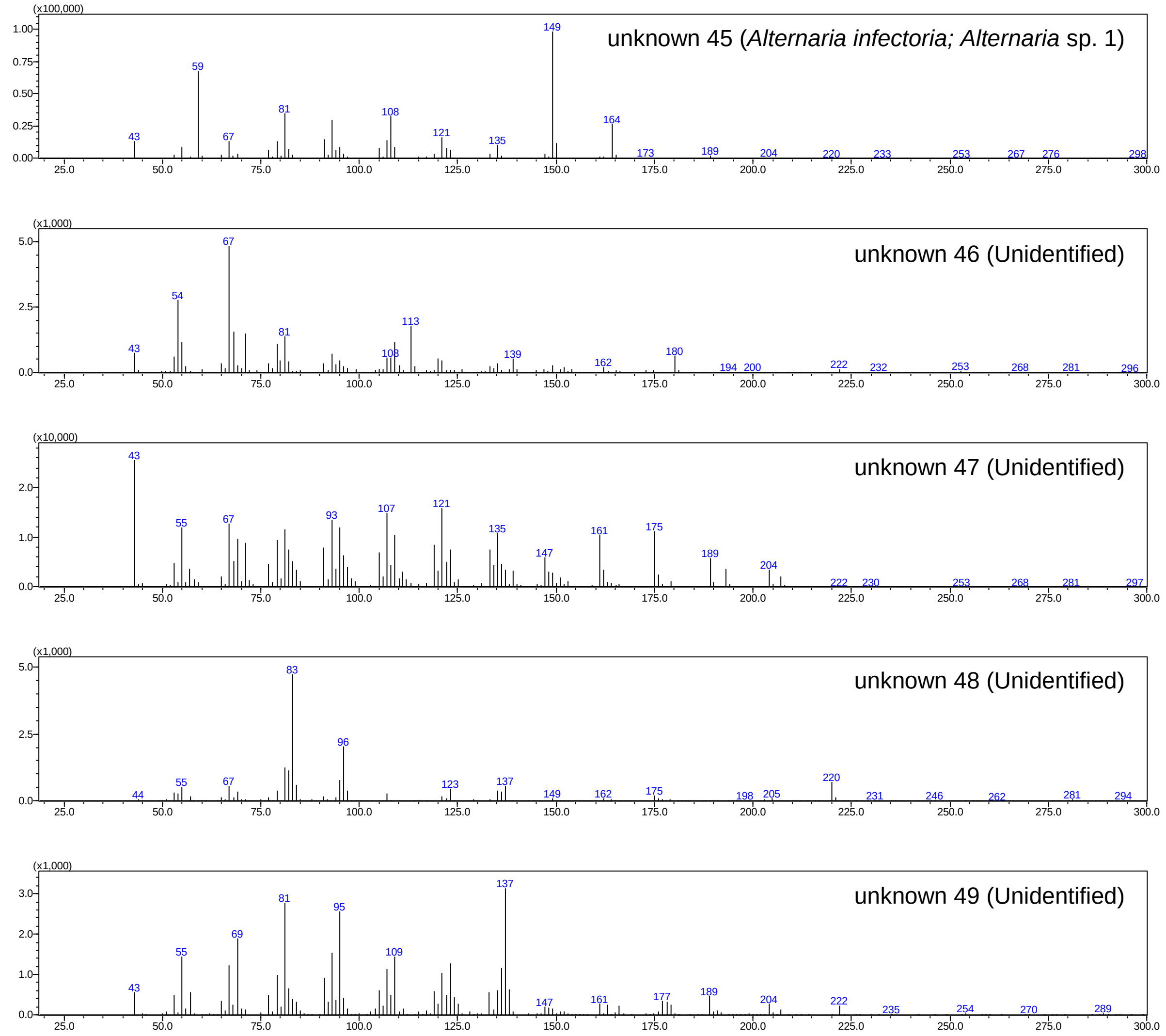

Figure S2: Mass spectra of unknown volatile organic compounds shown in Table 2. Background has been subtracted.

BUSCO version is: 3.0.2
The lineage dataset is: fungi_odb9 (Creation date: 2016-02-13, number of species: 85, number of BUSCOs: 290)
To reproduce this run: python /opt/software/bin/run_BUSCO.py -i /opt/software/packages/galaxy-dist/database/files/009/dataset_9623.dat -o busco_galaxy -l /opt/software/packages/busco/lineage/fungi_odb9/ -m transcriptome -c 11 -e 0.01 -z
#
Summarized benchmarking in BUSCO notation for file /opt/software/packages/galaxy-dist/database/files/009/dataset_9623.dat
BUSCO was run in mode: transcriptome

C:98.3%[S:93.1%,D:5.2%],F:1.4%,M:0.3%,n:290

285	Complete BUSCOs (C)
270	Complete and single-copy BUSCOs (S)
15	Complete and duplicated BUSCOs (D)
4	Fragmented BUSCOs (F)
1	Missing BUSCOs (M)
290	Total BUSCO groups searched

Figure S3: BUSCO analysis of the *Cladosporium* sp. *de novo* assembly. The BUSCO software tool [1] was used to validate the completeness of the *de novo* assembly.

Reference

1. Afgan, E.; Baker, D.; Batut, B.; Van Den Beek, M.; Bouvier, D.; Čech, M.; Chilton, J.; Clements, D.; Coraor, N.; Grüning, B. A., *Nucleic acids research* **2018,** *46* (W1), W537-W544.
